# The Long and Winding Road to Real-Life Experiments: Remote Assessment of Executive Functions with Computerized Games—Results from 8 Years of Naturalistic Interventions

**DOI:** 10.3390/brainsci14030262

**Published:** 2024-03-07

**Authors:** Melina Vladisauskas, Gabriel O. Paz, Verónica Nin, Jesús A. Guillén, Laouen Belloli, Hernán Delgado, Martín A. Miguel, Daniela Macario Cabral, Diego E. Shalom, Anna Forés, Alejandra Carboni, Diego Fernández-Slezak, Andrea P. Goldin

**Affiliations:** 1 Laboratorio de Neurociencia, Universidad Torcuato di Tella, Buenos Aires C1428BCW, Argentina; m.vladisauskas@gmail.com (M.V.);; 2 Consejo Nacional de Investigaciones Científicas y Técnicas (CONICET), Ministerio de Ciencia, Tecnología e Innovación, Buenos Aires C1425FQD, Argentina; 3 Centro de Investigación Básica en Psicología, Facultad de Psicología, Universidad de la República, Montevideo 11200, Uruguay; 4 Càtedra de Neuroeducació UB-EDU1st, Universitat de Barcelona, 08035 Barcelona, Spain; 5 Laboratorio de Inteligencia Artificial Aplicada, Instituto de Ciencias de la Computación, Universidad de Buenos Aires, Buenos Aires C1428EGA, Argentina; 6 Universidad de Buenos Aires, Facultad de Ciencias Exactas y Naturales, Departamento de Física, Buenos Aires 1428, Argentina; 7 CONICET—Universidad de Buenos Aires, Instituto de Física Interdisciplinaria y Aplicada (INFINA), Buenos Aires 1428, Argentina

**Keywords:** children, transfer, videogames, cognitive training, schools, child-ANT, Heart–Flower Stroop task, Corsi blocks, ToNI, Tower of London

## Abstract

Mate Marote is an open-access cognitive training software aimed at children between 4 and 8 years old. It consists of a set of computerized games specifically tailored to train and evaluate Executive Functions (EF), a class of processes critical for purposeful, goal-directed behavior, including working memory, planning, flexibility, and inhibitory control. Since 2008, several studies were performed with this software at children’s own schools in interventions supervised in-person by cognitive scientists. After 2015, we incorporated naturalistic, yet controlled, interventions with children’s own teachers’ help. The platform includes a battery of standardized tests, disguised as games, to assess children’s EF. The main question that emerges is whether the results, obtained with these traditional tasks but conducted without the presence of researchers, are comparable to those widely reported in the literature, that were obtained in more supervised settings. In this study, we were able to replicate the expected difficulty and age effects in at least one of the analyzed dependent variables of each employed test. We also report important discrepancies between the expected and the observed response time patterns, specifically for time-constrained tasks. We hereby discuss the benefits and setbacks of a new possible strategy for this type of assessment in naturalistic settings. We conclude that this battery of established EF tasks adapted for its remote usage is appropriate to measure the expected mental processes in naturalistic settings, enriching opportunities to upscale cognitive training interventions at schools. These types of tools can constitute a concerted strategy to bring together educational neuroscience research and real-life practice.

## 1. Introduction

In general, research methodology in cognitive neuroscience assumes that rigorously obtained lab results are comparable to what happens outside the laboratory, in ‘real life’. But this is not necessarily the case. The lab is quiet, stimuli are mostly isolated, instructions are straightforward, and, perhaps most importantly, the surroundings are novel and the subject knows it, possibly leading to more attentiveness, more alertness, and an increase in good-for-learning stress. Hence, how do we know if something works or does not work ‘for real’? An interesting approach is to run experiments in a familiar setting (to the subjects). In educational neuroscience, for instance, that might be to run experiments at schools. Furthermore, if experimenters were not even there, it would be almost as real world as it can get.

With this idea in mind, 15 years ago we developed Mate Marote, a free-to-access cognitive training software designed for children aged 4 to 8 years [1,2,3,4]. It comprises a suite of computerized games specifically tailored to training and assessing Executive Functions (EF). EF are a group of essential mental processes crucial for purposeful goal-directed behavior, which are typically described in the literature as comprising three core components: (1) an *inhibition* component, which enables individuals to resist strong inclinations in favor of more appropriate actions [5,6], (2) a *flexibility* component that pertains to the ability to shift between tasks or goals and adapt to a dynamically changing environment [7,8], and (3) a *working memory* component, involving the retention and mental manipulation of information [9,10]. These three core components are supported by *attentional processes* and are often referred to as “basic EF”, as other complex cognitive abilities build upon them, including *planning* and *relational thinking* [11,12].

In the literature, there are two important standardized batteries of evaluations designed to measure diverse cognitive aspects: the NIH toolkit and the Cambridge Neuropsychological Test Automated Battery [13,14]. Both include tests that target EF, besides language, memory, and processing speed. Neither of them is freely available for public use nor was tested in real-life settings. The NIH toolbox was originally validated as a desktop computer version used in the lab but, even though it was tested for its use on personal iPads (which is the actual modality), to our knowledge there are no reports of its application in naturalistic settings like children’s own classrooms with their usual devices and without the presence of researchers.

To provide a clear and broad picture of the general state of those aspects of children’s cognition, we built a battery employing versions of five cognitive assessments widely used in the literature that had already been standardized and validated. These include (1) the Corsi task, assessing visuospatial working memory [15,16], (2) the ToNI-4 task for relational thinking [17,18], (3) a Tower of London task for planning abilities [19], (4) the child-ANT task, measuring attentional networks [20], and (5) the Heart–Flower Stroop task, for assessing inhibitory control and cognitive flexibility [21].

Until 2015, interventions with Mate Marote were supervised, which means they required active participation from research assistants to provide individual instructions and to set the games participants would play in each session. Over the past eight years, we have implemented slightly different interventions, while maintaining the educational setting for all testing and training sessions. For instance, the software includes self-paced instructions for each game, and in each session the games are automatically enabled as appropriate. These adaptations were driven by the desire to scale up the number of children reached. In supervised interventions, a ratio of one to three students per teacher was required to play, while in the new ‘unsupervised’, or naturalistic, interventions, an entire classroom can participate in a session simultaneously, with less frequent need for an adult’s assistance.

While naturalistic interventions have replicated previous findings regarding children’s behavior during some Mate Marote games (e.g., [4,22,23]), cognitive tests were never assessed remotely. This study aimed to compare children’s results during naturalistic applications of a cognitive testing battery with the expected outcomes based on the existing literature for each task. These outcomes pertain to performance on a set of standardized cognitive evaluations incorporated as games into the Mate Marote platform to measure EF and attention. We hypothesized that performance in naturalistic settings would align with the expected patterns related to (1) the task-specific cognitive demand of the evaluations (the ‘difficulty effect’, based on each test’s design, e.g., [15,18,19,20,21]) and (2) the participants’ ages at the time of testing (the ‘age effect’, after [11,24]). Identifying these effects in naturalistic settings would bolster the presumption that remote interventions are as reliable as traditional in-person experiments, while affecting fewer personnel and demanding much less effort from educational institutions.

## 2. Materials and Methods

### 2.1. Participants and Procedure

A total of 605 children between the ages of 4 and 8 participated in this study, which spanned 24 interventions conducted between 2015 and 2023 in Argentina, Uruguay, and Spain (350 children on average were included in each task’s analysis; refer to Table S1 in the Supplementary Materials). These interventions occurred within children’s own educational settings and during regular school hours. The participation of the children was contingent upon written consent from their caregivers, and the study was conducted following the prior approval of an institutional ethical committee. Additionally, schools cooperated by providing the birth dates, genders, and diagnosed pathologies (when corresponding) of all participants. Birthdates and pathology data were not available for all the schools included in the study. No participants took part in more than one intervention.

Each of the cognitive training interventions pursued distinct objectives. For example, some aimed to compare training and control groups, while others sought to assess the efficacy of different training protocols (e.g., [3,22,25]). Regardless of their specific goals, all interventions started with one or two preliminary habituation sessions, in which children were familiarized with the platform, followed by two or three baseline testing sessions utilizing the Mate Marote software [1,26], after which each intervention continued at its own pace with its planned objectives. Children completed all tasks using the devices available at their institutions: laptops, PCs, or tablets. Each participant played on only one device each session, and teachers controlled that the answers were always given individually.

The focus of this study was on the results obtained after the baseline testing sessions. During this stage, all children completed a battery of standardized cognitive tests designed to evaluate EF and attention. The battery included five distinct computerized tests widely used in the cognitive evaluation literature:

1. The Corsi task [15].
2. The Test of Nonverbal Intelligence (version 4) [18].
3. A Tower of London task [19].
4. The child-ANT task [20].
5. The Heart–Flower Stroop test [21].

### 2.2. Description of the Computerized Cognitive Assessments

The five tasks that form this battery begin with a video tutorial where each game’s rules are explained, followed by a practice stage. During the latter, participants complete a set of 5 or 8 trials, depending on the task, and receive feedback on their responses. Every player must answer at least half of those correctly to move forward to the testing phase, and if that condition is not fulfilled the practice stage is repeated once. After two failed repetitions, the test begins its course anyways, and the player’s performance is discarded from the analysis. Performance on the practice trials is also excluded from the analysis.

#### 2.2.1. Tasks without Time Constraint

##### [1] Corsi Task

This test is widely utilized in clinical and research contexts to evaluate visuospatial working memory processes [27]. It entails participants reproducing a visual sequence in the exact serial order presented; the longer the sequence, the more challenging the trial.

**Figure 1:**
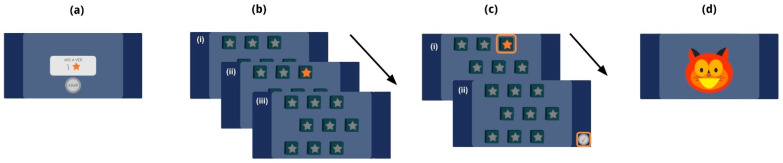
Sequence corresponding to a Corsi task trial. (**a**) Before the sequence to be recalled, the player is told its length (i.e., how many stars will shine, one in this example). When the player pushes the “start” button, the 9-fixed-stars board, based on the original design, appears. (**b**.i) The player starts by watching the fixed-stars board with no shining stars (1500 ms); (**b**.ii) a sequence of stars with a certain length shines, one at a time (for 2000 ms each), and (**b**.iii) after the last shining star turns off, the board is shown for 1500 ms, after which the player will try to mimic the previous sequence. (**c**.i) The player reproduces the sequence clicking over the stars in the same exact order as it was originally shown. (**c**.ii) Subsequently, the player clicks the check button (orange square on the bottom right). Arrows represent the automatic progress of the screens. (**d**) Nubis, the cat character, appears as a feedback sign on the screen accompanied with a sound, indicating whether the trial was correct (as shown) or not. The feedback is positive for a correct response and neutral for an incorrect one.

The computerized version of this task included in Mate Marote was previously employed in studies such as [26,28,29]. This version consists of a fixed configuration featuring 9 stars that intermittently illuminate in bright yellow and then extinguish (refer to Figure 1b). Importantly, the trials in this test do not impose a time constraint on players. Participants can take as much time as they require replicating the sequence and subsequently click the ‘check’ button for feedback, as illustrated in Figure 1c,d, respectively. As the test procedure unfolds, the number of stars within the sequences progressively increases, thus elevating the cognitive demand of each trial. The task starts with one star and concludes when a player provides three consecutive incorrect responses.

For each player, four accuracy measures were computed: (1) the number of stars of the longest correct sequence (i.e., the span), (2) the total number of correctly reproduced sequences, (3) the response time (RT) for each correct trial, and (4) the individual mean RT for each level of difficulty. The RT was normalized to log. A fifth accuracy measure, “score” (after Farrell Pagulayan and collaborators [30]), was computed by summing the stimulus length of every correct answer (for example, a player that answered correctly 2 sequences with length 2, and one with length 3, would have a final score of 2 + 2 + 3 = 7).

##### [2] Test of Nonverbal Intelligence

The Test of Non-Verbal Intelligence (ToNI), as described by [17], was developed to assess the ability of players to solve novel problems based on their capacity to perceive relationships and analogies, which is a measure of reasoning or relational thinking [31]. This test is designed to be suitable for populations whose verbal skills are still in the developmental stage, as it does not require explicit verbal abilities.

In each trial, two groups of abstract visual stimuli, typically geometric shapes or symbols, are presented in the “stimulus set” in the middle of the screen, and a set of options at the bottom. One shape is missing from the stimulus set and the task consists of identifying which of the options better completes the stimulus set. For that, players are tasked with identifying patterns and sequences and selecting the drawing that best corresponds to the recognized logical order. The assessment comprises up to 45 trials. The difficulty of each trial escalates by increasing the number of logical rules and the type of reasoning necessary to arrive at a solution [32].

**Figure 2:**
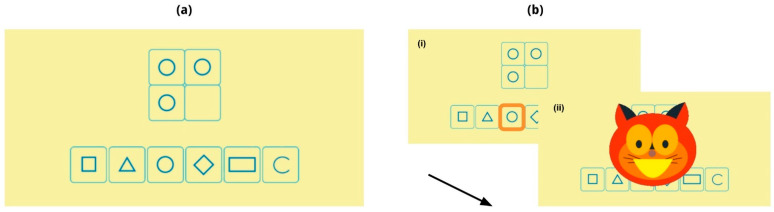
Sequence corresponding to a ToNI task trial. (**a**) The trial starts with a fixed stimulus set (above) and a set of options (below). The player must recognize that the relation between the figures in the stimulus set is that they are all the same geometric shape (circle) and choose, from the set of options, the missing figure that follows that logic. (**b**.i) The player chooses a figure from among the options (in this case, the circle highlighted in orange) and (**b**.ii) receives positive feedback from Nubis, the cat character. The feedback is positive for a correct response and neutral for an incorrect one. The arrow represents the automatic progress of the screens.

The example in Figure 2 shows the entire sequence of a ToNI task trial. The stimulus set in this case consists of 3 identical circles and the empty slot. Players must choose the missing drawing from the 6 options shown below. The correct response is the one that aligns most closely with the underlying pattern observed—in this case, the same geometric shape, highlighted in orange (example of a harder trial in Figure S1).

Participants are not subjected to time constraints for their responses. After arriving at a decision, they tap on the figure they deem correct. Importantly, whether the response is correct or incorrect, players do not receive feedback during the test. The termination criterion for this test is when a player provides three incorrect responses within five consecutive trials. Even though the task is not time-constrained, its duration did not extend beyond 6 min including the instructions.

For each player, two accuracy measures were computed: (1) the highest trial number completed correctly (i.e., the span), and (2) the individual RT for each trial. The RT was normalized to log.

##### [3] Tower of London Task

**Figure 3:**
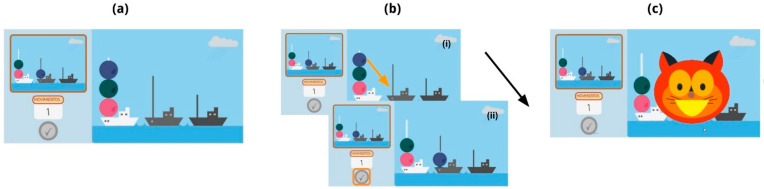
Sequence corresponding to a ToL task trial of the computerized gamified version of the test. (**a**) On the screen, the final configuration to be achieved is observed on the upper left, while the initial configuration that needs to be modified to reach the final configuration appears on the right. The figure also shows the different lengths of the rods, which can hold different numbers of balls: the shortest one, only one ball, the middle one, two balls, and the longest one holds all three balls. The desired number of movements is indicated below the final configuration (moving only one ball is sufficient to solve this trial). (**b**.i) The player moves the desired ball to an available spot using drag and drop (correct movement indicated with the orange arrow). (**b**.ii) When finished with the trial, the player clicks the check button on the bottom left, indicated with an orange square. The orange arrow represents the ball movement. The black arrow represents the automatic progress of the screens. (**c**) Correct feedback is received from Nubis, the cat character. The feedback is positive for a correct response and neutral for an incorrect one.

The Tower of London test (ToL), originally developed by Shallice [33], serves as a simplified adaptation of the Tower of Hanoi test [34]. Its primary aim is to assess planning processes. We designed a computerized version of the ToL test resembling the traditional manual version (see wooden artifact in Figure S2). This digital rendition also features two configurations, mirroring the elements in the traditional setup. These configurations encompass three balls each, as illustrated in the example trial in Figure 3.

The primary objective of this task is to transition from an initial configuration of balls on the rods to a specified final configuration. To accomplish this, the child is tasked with moving one ball at a time using drag and drop, while adhering to a predetermined minimum number of moves, which is provided at the outset of each trial. It is important to note that the child cannot move a ball to a rod that is already at full capacity. Specifically, the tallest rod can hold up to three balls; the middle rod can accommodate two, and the smallest rod, only one.

This test follows a structured format where each level of difficulty comprises a block of five trials, all of which share the same minimum number of moves required for successful completion. Similar to the Corsi and ToNI tasks discussed earlier, there is no time pressure associated with responding to each trial. Moreover, the complexity of the task escalates progressively as the trials advance. The degree of difficulty is associated with the number of moves necessary to transform the initial configuration into the final one. The test terminates if the child consecutively fails three trials in a row. One of the key performance metrics extracted from this task is the number of movements required to successfully complete the most challenging sequence, commonly referred to as the span. Also, like in the Corsi task, a player score was calculated by summing, instead of one point per trial, the difficulty level of every correct trial (see an example in Figure 1).

#### 2.2.2. Time-Constrained Tasks

##### [4] The Child Attentional Networks Task (Child-ANT)

The Child Attentional Network Test (ANT), an adaptation of its adult counterpart by Rueda and colleagues [20], was developed to evaluate three fundamental aspects of attentional processing in children, typically up to the age of around ten years. These aspects include alertness, orientation, and executive control. The shorter version of the task, used in this intervention, comprises a total of 64 trials, organized into two blocks, each containing 32 trials. Between the two blocks there is a pause screen, designed to prevent fatigue. When ready, participants have to push a “continue” button to indicate that they wish to continue playing.

The task requires children to determine whether a central animal is pointing to the right or left and then signify their choice by pressing the corresponding arrow on the screen. In each trial, the central animal is flanked by two additional animals on either side. These flankers can exhibit one of two conditions: they may either be oriented in the same direction as the central animal (congruent trial, as illustrated in Figure S3a above) or they may be oriented in its opposite direction (incongruent trial, as depicted in Figure S3a below). Generally, incongruent trials tend to be more demanding than congruent trials. To evaluate the executive attention network, the analysis involves subtracting the average RT (considering only correct trials) in congruent trials from those in incongruent trials.

**Figure 4:**
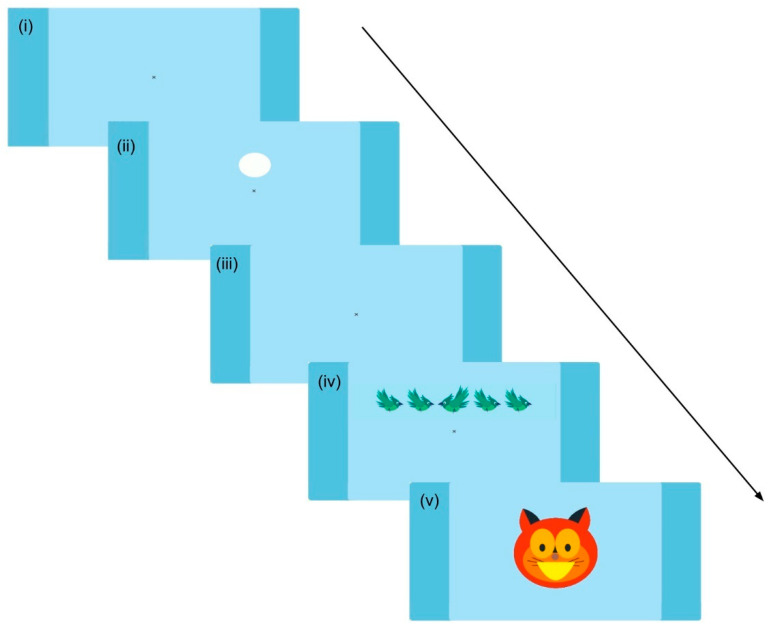
Trial sequence for the child-ANT task The arrow represents the automatic progress of the screens. (**i**) A fixation cross is displayed at the center of the screen for 1000 ms. (**ii**) One of the three spatial cues (or none, in the case of cue (**iv**)) appears for 150 ms. (**iii**) The fixation cross reappears for 450 ms. (**iv**) The stimuli (animals) appear on the screen and remain visible until the child responds, or for a maximum of 2500 ms. (**v**) Correct feedback is received from Nubis, the cat character, presented for 2000 ms. The feedback is positive for a correct response and neutral for an incorrect one. The interstimulus interval (ISI) is set at 1000 ms.

In 75% of the trials conducted, the screen presents one of three spatial cues 150 ms before displaying the animals (see a full incongruent trial sequence in Figure 4). These cues fall into three categories, described in detail in Section S2.2.2 in the Supplementary Materials and in Figure S3b: spatial cues (indicating where the target will appear), double cues (both above and below the center of the screen), and central (right over the fixation cross). The remaining 25% of trials do not include any cues. It is important to note that these cues are never explicitly explained to the children. Subtracting the performance on double-cued trials from trials with no cue reflects the alertness of the player (“alert attention network”). Comparing trials with a central cue to trials with a spatial cue allows estimation of the “orienting attention network”.

All trials are carefully counterbalanced to account for factors such as the conflict situation (the number of congruent and incongruent trials), the different types of cues, the positions of animals on the screen, and the side of the correct response. The primary outcome measures for this test include (1) the proportion of correct responses, calculated from the total number of trials answered (from now on referred to as “won”), and (2) the individual median RT, reflecting the time taken by each participant to respond to the various trials. For further details on the task, please refer to Section S2.2.2 in the Supplementary Materials.

##### [5] The Heart–Flower Stroop Test

**Figure 5:**
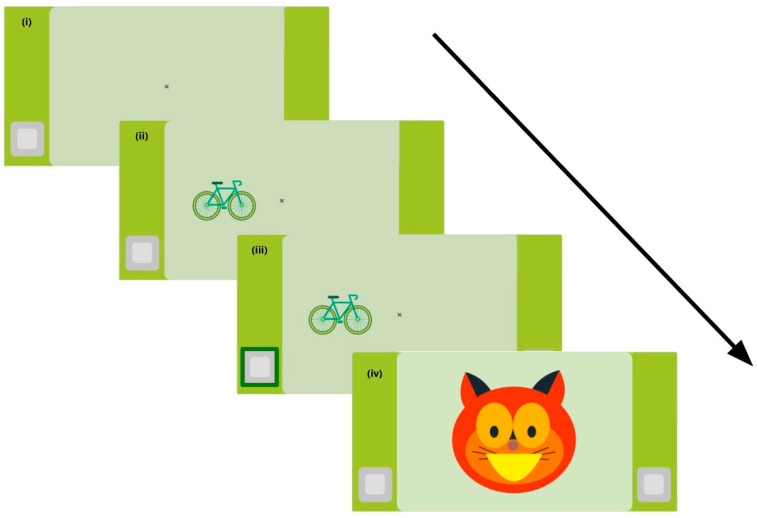
Trial sequence for the Heart–Flower Stroop task, the arrow represents the automatic progress of the screens: (**i**) starts with a fixation cross in the middle of the screen (1000 ms); (**ii**) followed by the stimuli (in this case, congruent, for up to 2500 ms); (**iii**) the answer is provided (in this case, correct); (**iv**) finally, correct feedback is received from Nubis, the cat character, presented for 2000 ms. The feedback is positive for a correct response and neutral for an incorrect one. The whole task includes counterbalanced left–right trials. The ISI is 1000 ms.

This adapted Stroop task (the Heart–Flower Stroop), developed by Davidson and collaborators [21], serves as an assessment tool for measuring inhibitory control and cognitive flexibility in children. In this task, participants are required to press one of two buttons based on the appearance and position of visual stimuli presented on the screen. The stimuli can be either a green bicycle or a pair of limes, positioned on either the right or left side of the screen. Crucially, in each trial, the stimulus and the correct response button can be on the same side (i.e., congruent trial) or on opposite sides (i.e., incongruent trial, see screenshots of each trial type in Figure S4). The full trial sequence for a congruent trial is depicted in Figure 5.

The task unfolds in three distinct stages. The initial phase comprises 12 congruent-only trials, enabling participants to become acquainted with the task. Subsequently, in the second phase, participants encounter 12 incongruent-only trials, where inhibitory control is crucial to provide correct responses. The final phase consists of 24 trials in which both congruent and incongruent trials are randomly counterbalanced. Participants must now demonstrate both inhibitory control and cognitive flexibility in this mixed-stage setting. Clear instructions and practice are provided before each stage to ensure comprehension. Specific instructions for each stage are administered immediately before it. To start playing after tutorials, participants must select a “play” button. This also allows them to rest between stages when needed.

Performance is assessed through two primary measures: accuracy, quantifying the number of correct responses in each stage, and RT, calculated as the median RT for correct responses, with responses under 250 milliseconds considered impulsive and excluded from analysis. To be included in the analysis, participants must meet a completion criterion of 50% in each test stage.

The primary outcome measures for this test include (1) the proportion of correct responses, calculated from the total number of trials answered (“won”, just like for the child-ANT task), and (2) the individual median RT, reflecting the time taken by each participant to respond to the various trials. The analysis process involves three key steps: first, comparing performance between the initial congruent-only and subsequent incongruent-only stages, to assess the ability to inhibit prepotent responses. Next, within the mixed stage, the same comparison is made to measure cognitive flexibility. Finally, a comparison is conducted between trial types of the same kind, but from different stages, shedding light on the additional cognitive flexibility demands that emerge in the last stage.

For further details on the task, please refer to Section S2.2.2 in the Supplementary Materials.

### 2.3. Testing Procedures

The whole battery of cognitive tests was administered in 2 sessions during the same week. Each session lasted no more than 20 min. The combination of tasks within each testing day varied between interventions, which might be a possible source of variability in the results (see Discussion section). Except for the ToNI task, pauses were included during each task to avoid fatigue and to ensure attentiveness. Corsi and ToL tasks include time to rest between trials (the child has to select a “play” button to see the stimulus). Child-ANT and Heart–Flower tasks have pauses within the full sequence (see details in each individual section above).

In each session, teachers logged in all children. During the interventions, there was no presence of investigators or research assistants at the schools and the instructions were provided by the software as appropriate. Children used, in every case, the devices available at their own schools to play. To control for novelty effects related to the devices, every participant went through a short habituation phase prior to the testing (see full description in [3]). For every task administered, instructions were provided by the software and an initial practice stage was completed and feedback was given to the participants on their performance (see Section 2.2 for further detail). Unlike supervised interventions, most of the classroom played simultaneously.

### 2.4. Paper’s Structure and Statistical Analysis

The primary objective of this study was to investigate whether a set of cognitive tests administered in naturalistic interventions would be sensitive to the expected age-and difficulty-related effects, rather than comparing the outcomes to specific benchmark values. The detailed hypotheses for each test, as well as for the composite cognitive performance score, are outlined in Table 1.

The results section of this paper is structured into four distinct subsections. The initial subsection encompasses descriptive statistics and fundamental demographic information regarding the dataset. The second subsection elucidates the findings related to the three tasks that do not impose time constraint, as elaborated in Section 2.2.1. The third subsection presents the outcomes of the two tests characterized by time constraint. The final section constitutes an analysis encompassing the entire battery of evaluations, culminating in an EF score computed as an average across different age groups. The following subsections outline the specific procedures employed for the analysis.

#### 2.4.1. Data Cleansing Process and Descriptive Statistics

Between the years 2015 and 2023, we performed a total of 10 interventions in school settings. Each intervention involved a varying number of participants belonging to different classes (for example, in some of the institutions the preschool students were distributed in classes A and B). In this analysis, every class was considered a subsample or “subset”. A total of 24 subsets were included in the analysis.

Initially, we performed outlier detection computing Z-scores for all pertinent response variables (see Table 2) within each task subset. Subsequently, we identified and excluded subjects with Z-values exceeding 3 or falling below −3. Following this initial exclusion process, we amalgamated the outcomes for each task across all subsets. The next step was to exclude the progress of subjects who presented any kind of pathology. Task-specific filters were also implemented later, aligning with baseline criteria outlined in their respective sections (Section 2.4.2 and Section 2.4.3).

To compute bi-variate correlations, we utilized the Spearman method from Scipy’s library and the partial correlation method from the Pengouin library, applying FDR (false discovery rate) corrections to account for multiple comparisons. The resulting correlations were visualized using Python 3’s Seaborn visualization library. All the outcome variables resulting from the interventions were included in the initial correlation analysis, but for the time-constrained tasks we just summed the performance on incongruent trials (see Section S2.4.1 in Supplementary Materials).

#### 2.4.2. Tasks without Time Constraint

Only participants who completed at least one trial correctly were included in the analysis. The primary outcome for individual participants encompassed three key variables:

1. Span: Gauges the difficulty of the most challenging trial successfully completed.
2. Score: The sum of the difficulty levels of all correctly answered trials.
3. Average RT: The mean RT of each difficulty level.

Span and score were Z- or log-transformed to normalize the corresponding distributions. For each of these response variables, we conducted linear mixed models using the Statsmodels Python library. These models incorporated subset as a random intercept variable to account for variations between interventions. Additionally, they included other pertinent explanatory variables as part of the fixed portion, depending on the analyzed task.

In each game, two relevant fixed variables were included: age and trial difficulty. Age was expressed as a continuous measure, in months (‘Age (cont)’). It was calculated based on the participant’s birth date and the month of the testing sessions. At times, for more precise developmental trends, this variable was categorized into 6-month groups (‘Age (cat)’). For instance, a child aged five years and one month was placed in the five-year-old group, while a child aged five years and six months was categorized in the five-and-a-half-year-old group. The difficulty variable changed for the different tests. For Corsi and ToL, trial difficulty was distinctly defined by the number of stimuli children had to either remember or plan. For the ToNI-4 task, trial difficulty was represented by the trial number.

Our model selection process followed a stepwise approach. Initially, a null model with only the random effects variable was run to establish baseline metrics. Subsequently, one explanatory variable was added at a time, resulting in models with the same complexity. We compared the latter to the null model using the likelihood ratio test, and all models that resulted in a significant better fit were compared by means of three key metrics: R-squared for mixed models (marginal R^2^ and conditional R^2^, following [35]) and the root minimum squared error (RMSE) [36]. This process was iteratively repeated, introducing additional explanatory variables to the best model from the previous step. A final model was chosen when the likelihood ratio test indicated no significant improvement in fit with increased complexity (for a similar methodology, see [4]).

Lastly, the performance of the chosen linear model was compared to logarithmic regression models using the same formula. These comparisons were also based on the RMSE, marginal R^2^ (which signifies the variance explained by the fixed effects), and conditional R^2^ (representing the variance explained by both the fixed and random effects). The best-fitting model among these alternatives was selected based on these three metrics, which are some of the most commonly used for selection across studies [37,38].

We applied the latter methodology to all the outcome variables in the time-unconstrained tasks, with the exception of the ToL. Due to technical issues, RT data were not trustworthy for this evaluation in the majority of the interventions (see Table S1). Consequently, no analysis related to RT could be run.

#### 2.4.3. Time-Constrained Tasks

The inclusion criteria for these tasks was achieving 50% completion (i.e., answered trials over the total amount of trials of the task). Also, since both tests include different stages or trial types, we only included participants who had completed at least one trial correctly from each category. The primary outcomes for individual participants were:

1. Percentage of Won Trials: The proportion of trials that a participant successfully completed, out of the total number of answered trials.
2. Median RT for Won Trials: This refers to the middle value of the RT recorded in successfully completed trials by each participant.

All variables were Z-transformed to normalize their distribution. Afterwards, the analytical approach mirrored the one described in the previous section, with one notable exception—the explanatory variable for difficulty. In the case of time-constrained tasks, difficulty was represented using categorical variables: “stimulus type” and “cue type” for the child-ANT task, and “stimulus type” and “block” for the Heart–Flower Stroop task (as detailed in Section 2.2.2).

To analyze the child-ANT task results, we calculated the outcome variables for the three attentional networks separately (as in Ref. [20]). Subtracting the performance of congruent from incongruent trials, we obtained both outcome variables for the executive network, Z-transformed. To obtain the alerting network values, we subtracted performance on double-cue trials from no-cue trials. Finally, orientation network outcome measures were calculated subtracting spatial cue trials from central cue trials. This procedure resulted in a total of 6 outcome variables related to the child-ANT task [20].

For the Heart–Flower Stroop task, we calculated both outcome variables to obtain two separate EF measures. To calculate inhibition abilities, we subtracted performance on the first block (only congruent trials) from the second block (only incongruent trials). To obtain cognitive flexibility outcome variables, we repeated the process but using incongruent and congruent trials within the third block, which had them mixed. This procedure resulted in a total of 4 outcome variables related to the Heart–Flower Stroop task [21].

#### 2.4.4. Composite Cognitive Performance Score

In order to facilitate performance comparison across tasks, to conduct a more in-depth analysis of age-related effects, following [39,40] we constructed a composite score for the entire battery of evaluations. To create the composite score, we first standardized the score of each task using a min–max scaler from the Scikit-learn library in Python. Standardization helps bring the scores from different tasks into a common scale, making them directly comparable. After standardization, we averaged the Z-scores for each child’s responses across tasks. It is important to note that we included only one variable per task in this composite score, and for that we chose the main performance metric in each case. For tasks without time constraints, we used the ‘span’ variable, while for tasks with time constraints, we used the won variable, both of which were related to accuracy.

We considered age as both a continuous and a categorical variable, as detailed in Section 2.4.2.

**Table 1:** Specific hypotheses for every task included in the tests battery. Details and explanations in Section 2.4.

Task Type	Outcome Measure(s)	Explanatory Variable(s)	Task(s)	Hypothesis
Time-unconstrained tasks	(1) Span (2) Score (3) Response time (RT) per difficulty level	(A) Age (cont) (B) Difficulty	Corsi task [15]	Older children are expected to show higher values of (1) and (2). Trials with higher amounts of stimuli to be remembered are expected to be harder and, therefore, show an increase in (3), and that effect is expected to be modulated by age.
			ToNI task [18]	Older children are expected to show higher values of (1) and (2). Trials with a higher amount of relations that have to be identified and integrated to respond correctly are expected to be harder and therefore show an increase in (3), and that effect is expected to be modulated by age [12].
			ToL task [19]	Older children are expected to show higher values of (1) and (2). Trials with higher amounts of movements required to solve the trial are expected to be harder and therefore show an increase in (3), and that effect is expected to be modulated by age.
Time-constrained tasks	(1) Proportion of correct answers (won) (2) Median RT for correct answers	(A) Age (cont) (B) Trial type	Child-ANT task [20]	Older children are expected to show higher values of (1) and lower values of (2). That effect is expected to be modulated by trial type, as follows: - incongruent trials should be harder than congruent trials. - trials without cue should be harder than the double-cued. - trials with central cue should be harder than the ones with spatial cues.
			Heart–Flower Stroop task [21]	Older children are expected to show higher values of (1) and lower values of (2). That effect is expected to be modulated by trial type, as follows: - incongruent trials should be harder than congruent trials. - stage 3 trials should be harder than respective trials from stages 1 and 2.
Composite cognitive performance score	(1) Average Z-score for accuracy	(A) Age	All of the above	Older children are expected to show higher (1).

**Table 2:** Number of participants overview. Initial participants: number of players who started the practice stage; outliers: number of participants excluded due to their extreme values (+/− 3 Z-score); excluded (pathology): number of children who were excluded from the analysis due to a known diagnosed pathology; excluded (failed): number of participants who were not included in the final sample due to specific criteria required for every task; final sample: final number of participants included in the analysis; available birthdate: birthdates were not available for every subset, so data from some participants were inferred by the school grade, but we were not able to calculate the exact age.

Task Type	Task	Initial Participants	Outliers	Excluded (Pathology)	Excluded (Failed Completion Criteria)	Final Sample (% Original)	Available Birthdate
Without time constraint	Corsi task	548	7	4	43	494 (90.14%)	422
	ToNI-4	480	14	4	11	451 (93.96%)	412
	Tower of London task	234	3	0	10	221 (94.44%)	211
Time-constrained	Child-ANT	384	8	4	23	349 (90.88%)	306
	Heart–Flower Stroop	488	4	4	28	452 (92.62%)	406

## 3. Results

### 3.1. Descriptive Statistics

More than 90% of participants completed the tasks, indicating that each game was well understood despite being administered in naturalistic settings (Table 2). It is important to note that, due to technical issues encountered in some institutions, not every intervention included all five of the tests described in this study. As a result, the sample sizes (N) varied for each test, which is reflected in the results presented for each task (Table 2).

To analyze the association among the different cognitive skills assessed, we built zero-order correlations and found distinct patterns between variable types across different tasks. We initially analyzed the relationship between all variables, and observed a high correlation among the attentional networks, with the executive being the most informative (see Figure S5). One prominent observation from the full correlation plot, after excluding orientation and alerting networks, is that similar variables tend to correlate with each other, demonstrating consistency in performance across tasks (Figure S6 and Table S2). This effect was found for Z-transformed won proportion, score, and response time outcome variables (ZWon, ZScore and ZRT, respectively). Also, as expected, lower correlation values are observed when examining the relationship between RT and accuracy variables (ZWon and ZScore). It is also noteworthy that the majority of them correlate with age.

**Figure 6:**
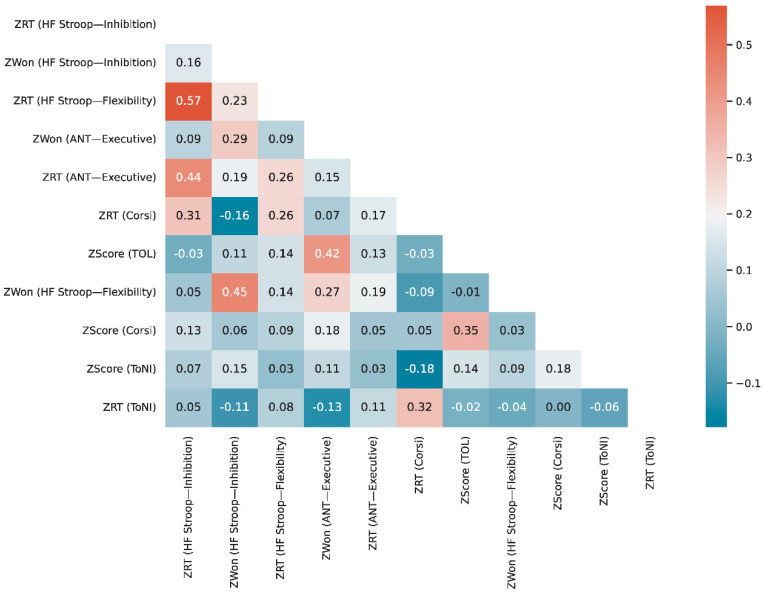
Heatmap depicting zero-order correlation between all measurements controlling by age. The most correlated variables are the ones that come from the same task or similar ones measuring accuracy or RT. Correlations are weaker when controlling by the age variable, as compared to the raw version (Figure S6).

After performing the pairwise comparisons, this time controlling for the contribution of the age variable, we observed that most of the correlations disappeared (Figure 6; for full correlation table and *p*-values, please refer to Table S3). This indicates that task results are mainly mediated by the participant’s age. The fact that the outcome variables are not correlated when the age variation is accounted for might reflect that the tasks succeed in capturing distinct cognitive constructs. Measures that result from the same evaluation still correlate to each other, as demonstrated, for example, by the 0.57 between ZRT for the two stages of the Heart–Flower Stroop task (noted as “Inhibition” and “Flexibility” in Figure 6 labels).

### 3.2. Time-Unconstrained Tasks

Full model formulas and statistical comparisons are available in Section S3.2 in the Supplementary Materials.

#### 3.2.1. Corsi Task

**Figure 7:**
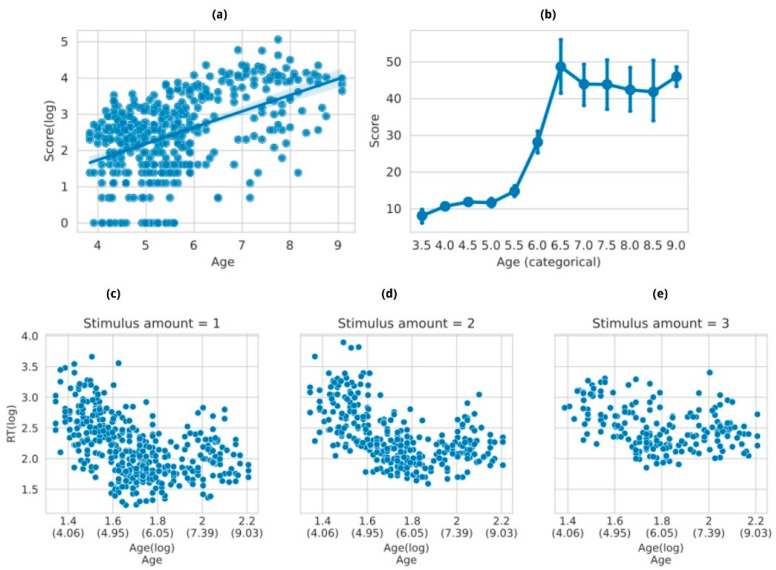
Corsi task score. (**a**) As expected, the working memory score increases with age. (**b**) Score performance across categorized age variable. When ages are collapsed every 6 months, a clear step in performance appears between 5.5 and 6.5, consistent with what is reported in the literature. (**c**–**e**) RT for Spans 1, 2, and 3 (from left to right). As expected, as children get older, they take less time to answer correctly. Also, as expected, as difficulty increases, so does the time they take to answer. The X axis depicts log-transformed age variable to reflect the best fit for these data.

The analysis for the working memory task included data from 422 children. As we hypothesized, based on the prior literature, the age effect was found to be significant across all models. Specifically, older children required less time to provide correct answers and reached higher scores, and this improvement appeared to stabilize with age (Figure 7). When age was analyzed as a categorized variable, an inflection point became evident between the performance of five and six-and-a-half year olds. Performance seemed to stabilize before and after this age range (see age distribution in Figure S7).

To statistically assess the relationship between the working memory score (log scale) and age, we employed the bottom-up model testing methodology outlined in Section 2.4.2. In short, we first compared a baseline model, with only subset as a grouping variable, to a model with age as the sole fixed effect. A likelihood ratio test confirmed that the addition of age improved the fit (χ^2^(1) *p* < 0.001). The linear regression resulted in a better fit for the data, explaining 42% of the total variance (Table S4 provides model estimates and *p*-values, and Table S5, the full model comparison). The ultimate model, represented as ‘Score (log) ~ Age + (1|Subset)’, confirmed our expectations by revealing a significant predictive relationship between the age variable and performance in the Corsi task. Specifically, age exerted a noteworthy influence on the logarithm of score (χ^2^(1) = 32.55, *p* < 0.001), resulting in an average increase of approximately 2.7 ± 0.4 (SE) (15 points in the original scale). This means that visuospatial working memory improves at these ages slowly, yet steadily, as reported in the literature.

To analyze to what extent age affected speed performance (RT), we conducted separate linear mixed models for stimulus amounts of 1, 2, and 3 items (Figure 7c–e). Our decision to limit the regression analysis to answers up to a span of 3 was based on the observation that younger children were unable to reach more demanding trials. Unlike the working memory score analysis, results in this case indicated that a logarithmic regression model provided the best fit for the data, as suggested by the patterns seen in Figure 7 (see full model comparison in Table S5). As expected, the logarithmic transformation of age was associated with a reduction in RT. For stimulus amount 1, the RT (log) decreased by −0.898 ± 0.202 (SE). When translated to the original scale, this suggests that, on average, RT is expected to decrease by approximately 40.6% for each unit increase in age, with a 95% confidence interval ranging from a 29.8% to a 55.3% reduction. All three models demonstrated superior predictive capabilities for the data, as reflected in their marginal and conditional R^2^ values, which ranged from 0.30 to 0.70. Table S6 summarizes the parameters for the best models in each case.

Difficulty effect was confirmed as well, after analyzing all stimulus amounts together using RT (log) as the outcome variable in the model ‘RT (log) ~ Age + Stimulus amount + (1|Subset)’ (see full model output in Table S7).

#### 3.2.2. ToNI Task

Similar patterns to those observed in the Corsi task were evident in the relational thinking test. A total of 451 participants passed the inclusion criteria and were therefore included in the analysis, and birthdates were available for 412 of those participants (see age distribution in Figure S8). Due to technical issues, RT data were not trustworthy for interventions from 4 to 12 (see Table S1) and the analysis was performed with the remaining information.

**Figure 8:**
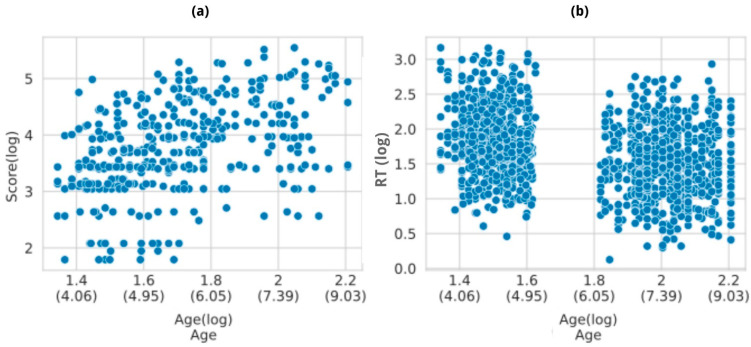
(**a**) Score regression on the ToNI task. As expected, the relational ability measured with this task increases with age. (**b**) RT distribution according to age. As observed in the Corsi task, the time children take to correctly answer decreases with age. The age gaps are due to the absence of subjects aged between 5 and 6 years old (see Table S1). In both cases, the X axis depicts the log-transformed age variable to reflect the best fit for these data.

Age was found to be a significant explanatory variable for both score and RT (when applicable), both in a log scale (Tables S8 and S9), as suggested by the trends depicted in Figure 8. The best fit that captured the impact of age on the level of difficulty achieved was a logarithmic model (RMSE = 0.704 and conditional R^2^ = 0.228, complete model output in Table S8). For the RT response variable, we performed the bottom-up model selection procedure described in Section 2.4.2. In this case, both response variables improved the model fit compared to a null model (see Table S9 for model output). After comparing to a logarithmic regression, the best model turned out to be: ‘RT (log) ~ Age (log) + Stimulus Amount + (1|Subset)’ (detailed model comparison in Table S10). The latter predicts that RT reduces as age increases with a rate of −0.765 ± 0.117 (SE), which in the original scale is a 47.8% decrease for each unit increase in the logarithm of age (approximately 2.718 years), with a standard error of 11.7%. Furthermore, the difficulty effect was also confirmed, as the trial number variable showed a positive effect on RT, with a change of 0.724 ± 0.036 (SE). This implies that, on the original scale, RT is expected to increase by about 106.3% for each unit that the logarithm of trial difficulty increases (almost three difficulty levels higher), with a standard error of 3.6%.

#### 3.2.3. ToL Task

As described in Section 2.4.2, the RT outcome variable was not analyzed for this task (see age distribution for the 211 included participants in Figure S9).

**Figure 9:**
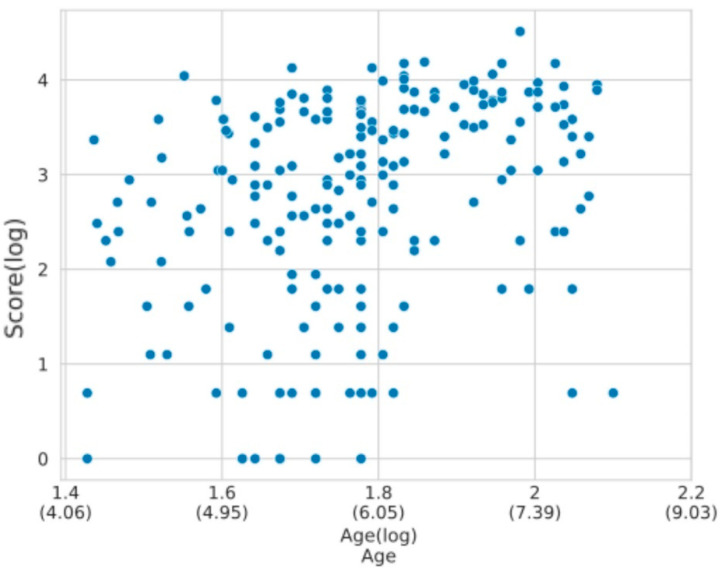
Score regression on the ToL task. As expected, the planning ability measured with this task increases with age. The X axis depicts the log-transformed age variable to reflect the best fit for these data.

We first constructed a baseline model and compared it to one including the age variable as a predictor, ‘log(Score) ~ Age + (1|Subset)’, which resulted in a better fit for the data (χ^2^(1) = 10.03, *p* < 0.001). The best model fit for the data was a logarithmic regression (Tables S11 and S12 for the full model output). The difference between conditional and marginal R^2^ values (41% vs. 22%, respectively), suggests that the random intercept associated with the subset variable (i.e., the differences between interventions) plays an important role in the observed outcomes of this task. Despite the suboptimal fits for the fixed portion of the model, the age (log) variable remained a significant explanatory factor (Figure 9). This result reinforces our main hypothesis that the expected effects are consistently observed in naturalistic settings.

### 3.3. Time-Constrained Tasks

Two tasks requested participants to respond to each trial before a certain amount of time. Full model formulas and statistical comparisons of both are available in Section S3.3 in the Supplementary Materials.

#### 3.3.1. Child-ANT Task

As the child-ANT task comprises a fixed number of trials that every participant is expected to complete, performance in this task cannot be assessed using a score measure. Rather, it is analyzed based on the won and median RT outcome variables for each player. In this task, trial difficulty is represented by the stimulus type and the cue type variables (see Section 2.2.2, “child-ANT task”). In short, incongruent trials are expected to be more difficult than congruent trials, and trials with no informative cues are expected to be more difficult than those with informative cues.

**Figure 10:**
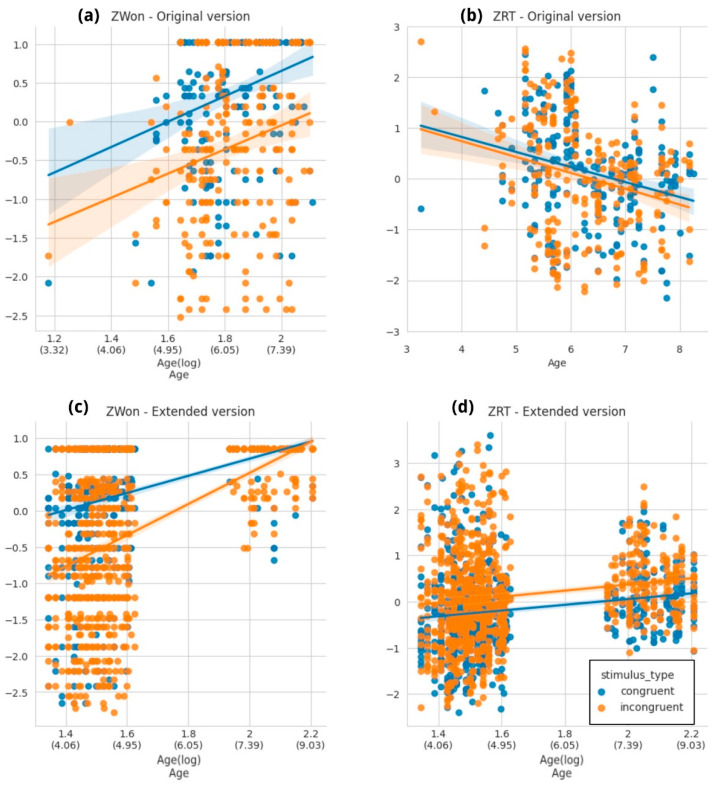
Performance for the original (**a**,**b**) and the extended (**c**,**d**) versions of the child-ANT **task.** Each dot corresponds to a child’s proportion of correct trials (**a**,**c**) or individual median of RT (**b**,**d**). In blue: congruent trials; in orange: incongruent trials. (**a**) ZWon for the original version of the child-ANT task across ages. As expected, children are better at solving congruent trials and, regardless of the stimulus type, their ability to respond correctly increases with age. The X axis depicts log-transformed age variable to reflect the best fit for these data. (**b**) ZRT for the implementation of the child-ANT task in its original form. There are no differences along the age curve nor between trial types. (**c**) ZWon for the extended version of the child-ANT task across ages. As expected, children are better at solving congruent trials and, regardless of the stimulus type, their ability to respond correctly increases with age. The X axis depicts log-transformed age variable to reflect the best fit for these data. (**d**) ZRT for the extended implementation of the child-ANT task. Incongruent trials take longer to be solved, but there are no differences along the age curve. The X axis depicts log-transformed age variable to reflect the best fit for these data. (**c**,**d**) Age gaps are due to the absence of subjects aged between 5 and 6.5 years old. Full model comparisons available in Tables S17 and S18.

In the initial naturalistic interventions, we observed a high proportion of unanswered trials (see Figure S10). Consequently, we decided to implement an extended version of the task giving 5000 ms to answer. Since the task with the original trial duration (i.e., 2500 ms) is the most commonly applied in the literature, we conducted the planned analysis separately for both configurations: the original trial duration (N = 99) and the extended trial duration (N = 207), as shown in Figure 10 (original version in the top row, extended version at the bottom). The age distributions for both versions are in Figure S11.

Overall, the expected difficulty effect was present in association with the stimulus type variable in both the extended and original versions (Figure 10a,c), indicating that participants took longer to answer incongruent trials and also made more mistakes. The only exception was in the original version, in which no difference between trial types was observed for ZRT (Figure 10b). Cue type was not a powerful explanatory variable in any of the models describing the outcomes of the task, which was unexpected given that this evaluation was designed to compare performance between different types of trials (see Discussion Section 4.2).

For the original trial version, age was only a good predictor when ZWon was the response variable (Table S13), indicating, as expected, that as children become older the proportion of correct answers increases (Figure 10a). The conditional R^2^ indicates that the model explains only 21% of the variance; hence, there must be other unaccounted for factors influencing these outcomes such as interindividual differences. The model with RT as the response variable resulted in an even poorer fit (Table S14); after comparing the null model to three models with one explanatory variable each, none improved the model significantly after a likelihood ratio test (age: χ^2^(1) = 0.163, *p* = 0.685; cue: χ^2^(1) = 0.304, *p* = 0.581; stimulus type: χ^2^(1) = 1.094, *p* = 0.295).

In the case of the extended version, as mentioned before, the expected significance of age and stimulus type variables was observed both for the ZWon and the ZRT variables (see Tables S15 and S16). The best fit in both cases was a logarithmic model, but in terms of the RT variable, marginal and conditional R^2^ values explain less than 10% of variance. Furthermore, the age effect is present, but with an opposite slope compared to our expectations (0.611 ± 0.187 (SE)).

#### 3.3.2. Heart–Flower Stroop Task

**Figure 11:**
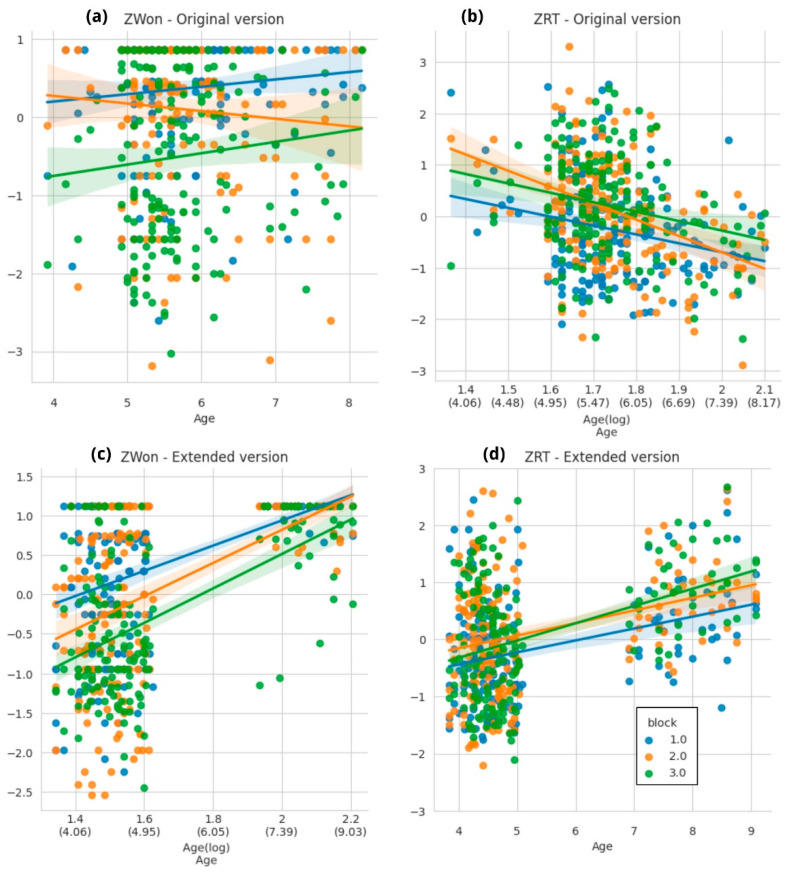
(**a**) ZWon for the original version of the Heart–Flower Stroop task across ages. Children are better at solving congruent trials as expected, and regardless of the stimulus type the ability to respond correctly increases with age. There is an interaction between age and stimulus type, where both congruent and incongruent trials are answered correctly in a very high proportion by older children. (**b**) RT median for the original implementation of the Heart–Flower Stroop task. Incongruent trials take longer to be solved, but there are no differences along the age curve. The X axis depicts log-transformed age variable to reflect the best fit for these data. (**c**) ZWon for the extended version of the Heart–Flower Stroop task across ages. Children are better at solving congruent trials as expected, and regardless of the stimulus type the ability to respond correctly increases with age. There is an interaction between age and stimulus type, where both congruent and incongruent trials are answered correctly in a very high proportion by older children. (**d**) RT median for the extended implementation of the Heart–Flower Stroop task. Incongruent trials take longer to be solved, but there are no differences along the age curve. The X axis depicts log-transformed age variable to reflect the best fit for these data. (**c**,**d**) Age gaps are due to the absence of subjects aged between 5 and 6.5 years old. Full model comparison available in Tables S23 and S24.

Similar to the child-ANT task, during the first naturalistic implementations of this test we encountered a significant amount of non-responded (i.e., missed) trials. So, in this case also we decided to explore an extended version in which participants had 2500 ms more to answer each trial. We performed the analysis separately for both configurations: the original trial duration (N = 211) and the extended trial duration (N = 192; see Figure 11). The age distributions for both versions are in Figure S12.

In the original implementation, the ZWon variable was only influenced by block, as predicted by a linear mixed regression (Figure 11a, model output available in Table S19). Contrary to expectations, age was not a significant predictor, probably due to the high proportion of missed trials (see further analysis in the Discussion section).

In the extended version, the same regression model resulted in a better fit (explaining 32.4% of the variance), and both the explanatory variables and their interaction were significant predictors. These results are more in line with reports from past implementations of this task, as reported in the literature [21,29] (see model output in Table S20).

In the original version of the task, the expected RT trend is observed, with age and block as significant predictors. However, in the extended version, results are similar to those reported for the child-ANT task: the model suggests that block is not a good predictor, and age affects RT in a way opposed to what was predicted, that is, as children grow older, they take more time to answer (see Tables S21 and S22 and Figure 11c,d).

### 3.4. Composite Cognitive Performance Score

**Figure 12:**
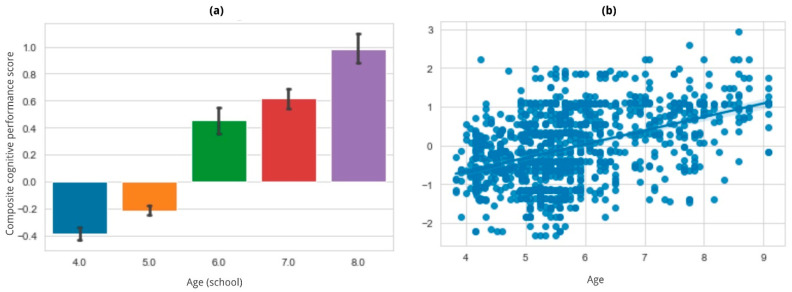
The composite cognitive performance score plotted according to (**a**) school age, (**b**) continuous age. As expected, the Z-score increases with age.

To condense all measurements to encompass an ‘overall cognitive performance outcome’, we constructed a composite by averaging the performance across the five individual tasks (see Section 2.4.4 for further details on the methodology). The composite was regressed on an indicator of the child’s age (either continuous, calculated based on the birth date, or discrete based on the school age) and included the intervention as the random portion of a linear mixed model. In both cases, in line with our hypothesis, the score was significantly predicted by age (see Figure 12), indicating a strong correlation with overall cognitive performance. Both model estimates and *p*-values are presented in Tables S25 and S26.

## 4. Discussion

In this article, we present results obtained in naturalistic settings obtained over 8 years. We collected data from a total of 24 different interventions inside schools, involving 605 children aged 4 to 8 years old in three different countries, and assessed their performance on a computerized battery of gamified standardized EF tests. Contrary to the usual educational neuroscience interventions, we here show that reliable measurements can be obtained in typical classroom environments and without the presence of researchers, as data were collected simultaneously in entire classes. Therefore, we confirm this approach can be reliably used to study cognition and development in naturalistic settings.

Overall, we hypothesized that children’s performance on these tasks should be comparable to what is described in the literature, although not necessarily identical (Table 1). We evaluated the presence of two widely reported effects: (1) a difficulty effect, related to the cognitive demands of each test, and (2) an age effect. Results indicate that children’s EF performance in naturalistic interventions aligns with what is reported in the literature, as both predicted effects were consistently observed in every task. Furthermore, they were captured by a unique measurement that condensed general cognitive performance.

### 4.1. Time-Unconstrained

Three of the tasks included in the battery allowed players to take their time to answer every trial. In these, the duration of the whole task was not fixed but performance-dependent.

The Corsi task measures spatial working memory. The literature reports that working memory span tends to increase with age, especially between 5 and 6 years old [41]. Even though age was found to be a significant predictor of performance, we did not observe this effect beyond the age of 6. This could be due to a reduced number of older participants, or to the possibility that this task in naturalistic settings is more informative for younger children. Our findings align with another study [30], albeit one using a manual version of the task, which describes a plateau in working memory span between 7 and 8 years old. Regarding RT, the performance distribution for each difficulty level appeared logarithmic, with the best fit to a logarithmic regression curve observed in trials requiring the recall of three stimuli. This indicates a clear difficulty effect related to the number of stimuli children need to remember to respond accurately, as expected.

The ToNI-4 and Tower of London tasks, which assess relational and planning skills, respectively, encompassed smaller sample sizes due to technical difficulties we encountered. Despite this limitation, some conclusions can be drawn from the children’s performance. In the ToNI-4 task, a logarithmic model was the best fit for the performance curve, indicating significant effects of age and difficulty. The score variable was significantly predicted by age, with a logarithmic mixed regression as best fit, explaining a substantial portion of the variance, including a considerable contribution from each intervention (the random portion of the model).

The Tower of London task exhibited the predicted age effect. Probably, given the small sample size, the effect was only moderately significant and the marginal R2 indicated that less than 25% of the variance was explained by the best fit (logarithmic mixed) model. Together with the ToNI-4 score variable model, the two tests showed the poorest marginal R2. Interindividual differences were noticeable, as shown in Figure 8a and Figure 9, which might be causing the poor fit due to unaccounted-for variability. Additionally, including RT data in the analysis may provide a more comprehensive understanding of the expected effects, in alignment with the literature on this task [42,43].

The logarithmic regression was the best fit for all the analyzed variables resulting from time-unconstrained tasks, which might suggest that the performance improvement is not occurring at the same rate across the whole age curve, but somehow ‘slowing down’ when children get older. This appears to be evident in both RT and score variables. The difficulty effect, related to the “stimulus type” and “trial number” variables in the Corsi and ToNI-4 tasks, respectively, was confirmed as expected, therefore contributing to the idea that time-unconstrained tasks work similarly in naturalistic interventions to how they have been reported in the literature for typical interventions.

### 4.2. Time-Constrained Tasks

Two of the tasks in the battery were time-constrained: the child-ANT, designed to measure attentional networks, and the Heart–Flower Stroop task, designed to assess inhibition and cognitive flexibility. Initially, the first naturalistic interventions using these tasks resulted in a higher-than-expected proportion of missed trials. To address this issue, the available RT for each trial was extended from 2500 to 5000 ms. In this study, we analyzed the results of both versions separately and assessed whether the expected age and difficulty effects were observed in each case.

For the child-ANT task, both age and difficulty effects were observed in each trial version. However, there was a more pronounced ceiling effect in the extended version, suggesting that, as expected, the task is easier with more available time to respond. This ceiling effect could be causing the significant interaction between stimulus type and age because, for older children, the task gives enough time to demonstrate an almost perfect performance. Regardless of this, in both versions the proportion of correct trials over the total amount of answered trials was used as the accuracy outcome variable. It is worth noting that results for the original (shorter) version were suboptimal, as near 10% of the explained variance related to the best model fit using ZWon as the outcome variable.

In contrast to our hypotheses, the results for RT were remarkably different. For the original version, children’s age was not a good predictor of RT, while for the extended version it was, but in an unexpected direction (higher RT for older children, Figure 10d). The models poorly fit the data in both cases, with low percentages of explained variability, as indicated by the marginal and conditional R^2^. Regardless of the poor fit, there are some reports in the literature showing that as children get older they slow down as a strategy to preserve accuracy [21,44,45]. Hence, it is possible that due to the time extension older participants might be realizing that they can slow down to be more precise, which may also explain the ceiling effect observed in accuracy.

The child-ANT original paper [20] reported specific performance results for children within the age range included in this study. These results showed that age was not a good predictor of RT after analyzing each network independently, which was one of the main expected effects of this study. Therefore, it is possible that even in more controlled interventions, RT might not be an informative measure of attentional networks, at least for this age range, and that this outcome in naturalistic settings is not an exception. Consequently, we recommend using the extended version to reduce the risk of a high proportion of missed trials, as observed in our earlier interventions using the original version.

The assessment of the Heart–Flower Stroop task yielded similar outcomes to the child-ANT task. It comprises three task block types (congruent, incongruent, and mixed) which represent different difficulty levels. This was reflected in the performance obtained after both task versions (original and extended), although in the original version using ZRT as the outcome variable block was not a significant predictor. The age effect, however, was present in both outcome variables for the extended version of the task, even though the age range between 5 and 6 years old was missing. The original version of the task in naturalistic settings appeared to elicit greater variability than usual, possibly contributing to the small difficulty effect and the absence of the age effect using ZWon as the outcome variable.

The extended version of the Heart–Flower Stroop task, however, did not yield the expected effects in RT. Unlike the child-ANT case, for this task, the original paper [21] indicated a difference in RT for the age ranges considered in this study. Specifically, older children slowed down on the harder trials (i.e., the mixed condition), allowing them to maintain accuracy, while younger subjects responded more consistently and impulsively across conditions. Thus, contrary to expectations, a decrease in RT is not always observed across the age curve, and so our results could be reflecting that reported effect. RT appears to be a more challenging measure to interpret, which might be due to the high intersubject variability; hence, we suggest continuing to use the extended version of the task to evidence the more consistently reported won difference between blocks.

### 4.3. General Discussion: Strengths and Limitations

The tasks employed in the 24 interventions included in this study positively align with the results reported in the literature, as the expected age and difficulty effects were observed in at least one of the dependent measures for each task. The averaged accuracy results also indicate a strong and significant age effect, confirming that the battery is sensitive to expected developmental changes in the measured cognitive processes, which is in line with typical supervised interventions.

The presence of the two evaluated effects (age and difficulty) in at least one of the dependent variables for every test suggests that the battery is suitable for its intended purpose, even in naturalistic settings, which differ significantly from the original controlled settings. Our study also recommends the continued use of the extended version of time-constrained tasks (i.e., the child-ANT and the Heart–Flower Stroop tasks) to prioritize the accuracy variable, which appears to be more informative than RT in capturing developmental trends. In this sense, the composite score capturing the essence of this aspect of cognitive performance can constitute a good approach to analyze overall executive function status.

We acknowledge that there are several aspects to consider when interpreting these analyses. One important factor is the diversity of the sample, which includes interventions conducted in different regions and countries (Argentina, Uruguay, and Spain). Additionally, a socioeconomic status variable was not included in the analysis, which could potentially account for some of the high intersubject variability observed. Future studies could benefit from including standardized questionnaires to assess this variable and provide a more comprehensive analysis [29]. A crucial point to highlight is that the children were playing in their everyday school settings for this study, with only their teachers as monitors, and with the equipment available at their schools (which, most of the time, are not the best devices, nor do they have the best connectivity). And, yet, we still succeeded in seeing effects. This is undoubtedly a strength of our study.

## 5. Conclusions

During the course of 8 years and 24 interventions inside schools, we collected data from 605 4-to-8-year-olds in three different countries. In this manuscript, we prove that the computerized battery of gamified standardized EF tests that are included in the Mate Marote platform can effectively, and reliably, assess cognitive performance in a typical classroom environment, and without the presence of researchers. Results obtained inside the schools, including a unique condensed measurement of cognitive performance, align with what was expected from the literature.

In conclusion, the findings from this study are encouraging, as naturalistic interventions offer the advantage of collecting a larger amount of data from many children simultaneously and outside the lab, in naturalistic settings, paving the way to more real-life-situated research.

## Data Availability

The data that support the findings of this study are available from the corresponding author upon reasonable request. The data are not publicly available due to privacy concerns.

## Supplementary Materials

The following supporting information can be downloaded at: https://www.mdpi.com/article/10.3390/brainsci14030262/s1.
