# Supplementary material for: The Long and Winding Road to Real-Life Experiments: Remote Assessment of Executive Functions with Computerized Games—Results from 8 Years of Naturalistic Interventions
Source: Brain Sci. 2024 Mar 7;14(3):262. doi: 10.3390/brainsci14030262 (PMC10968583; doi:10.3390/brainsci14030262)
Supplement: Supplementary file 1 [file brainsci-14-00262-s001.zip › brainsci-2820109-supplementary.pdf]

brain sciences

MDPI

Supplementary material

The Long and Winding Road to Real Life Experiments. Remote Assessment of Executive Functions with Computerized Games: Results from 8 Years of Naturalistic Interventions

Melina Vladisauskas[1,5], Gabriel Paz[1,5], Verónica Nin[3], Jesús A. Guillén[2], Laouen Belloli[4,5], Hernán Delgado[3], Martín A. Miguel[4,5], Daniela M. Cabral[1,4], Diego E. Shalom[6,7], Anna Forés[2], Alejandra Carboni[3], Diego Fernández-Slezak[4,5], Andrea P. Goldin[1,5,*]

[1] Laboratorio de Neurociencia, Universidad Torcuato di Tella, Buenos Aires, Argentina; m.vladisauskas@gmail.com (M.V.); (G.P.); (D.M.C.); (A.P.G.)

[2] Cátedra de neuroeducación ubadu1st, Universidad de Barcelona, Barcelona, España; (J.A.G.); (A.F.)

[3] Centro de Investigación Básica en Psicología, Facultad de Psicología, Universidad de la República, Montevideo, Uruguay; (V.N.); (H.D.); (A.C.)

[4] Laboratorio de Inteligencia Artificial Aplicada, Instituto de Ciencias de la Computación, Universidad de Buenos Aires, Buenos Aires, Argentina; (L.B.); (M.A.M.); (D.F.S.)

[5] Consejo Nacional de Investigaciones Científicas y Técnicas (CONICET), Ministerio de Ciencia, Tecnología e Innovación, Buenos Aires, Argentina

[6] Universidad de Buenos Aires, Facultad de Ciencias Exactas y Naturales, Departamento de Física, Buenos Aires, Argentina; (D.E.S.)

[7] CONICET, Universidad de Buenos Aires, Instituto de Física Interdisciplinaria y Aplicada (INFINA), Buenos Aires, Argentina

* ***Correspondence: andrea.goldin@utdt.edu***

Section 2.1. *Participants and procedure.*

Table S1. Detailed description of the interventions included in the study and the participants that took part in each one of them.

Intervention n°	Year, Month	Country	Scholar age	Average age (SD)	Gender proportion (N_{girls})	Initial participants (total)	Completed tasks (N)
1	2015	Argentina	5	Missing data	0.47 (23)	49	Child-ANT<br>Stroop<br>Corsi
2	2016	Argentina	6	6.70 (0.31)	0.55 (15)	27	Child-ANT<br>Stroop<br>Corsi<br>ToNI<br>ToL**
3	2016	Argentina	7	7.22 (0.34)	0.4 (12)	30	Child-ANT<br>Stroop<br>Corsi<br>ToNI<br>ToL**
(cont. in next page)							

brain sciences

MDPI

(cont. Table S1)

4	2016, April	Uruguay	5	5.41 (0.28)	0.44 (11)	25	Stroop<br>Corsi<br>ToNI*<br>ToL**
5	2016, April	Uruguay	5	5.42 (0.27)	0.46 (13)	28	Stroop<br>Corsi<br>ToNI*<br>ToL**
6	2016, April	Uruguay	5	5.26 (0.34)	0.43 (9)	21	Stroop<br>Corsi<br>ToNI*<br>ToL**
7	2016, April	Uruguay	5	5.09 (0.48)	0.34 (10)	29	Stroop<br>Corsi<br>ToNI*<br>ToL**
8	2016, October	Uruguay	5	5.88 (0.28)	0.41 (7)	17	Stroop<br>Corsi<br>ToNI<br>ToL**
9	2016, October	Uruguay	5	5.86 (0.25)	0.45 (9)	20	Stroop<br>Corsi<br>ToNI*<br>ToL**
10	2016, October	Uruguay	5	5.90 (0.31)	0.43 (9)	21	Stroop<br>Corsi<br>ToNI*<br>ToL**
11	2017, April	Uruguay	5	5.20 (0.47)	0.47 (7)	15	Stroop<br>Corsi<br>ToNI*<br>ToL**
12	2017, April	Uruguay	5	5.33 (0.30)	0.68 (13)	19	Stroop<br>Corsi<br>ToNI*<br>ToL**
13	2020	Spain	5	5.61 (0.28)	0.4 (10)	25	Child-ANT<br>Stroop<br>Corsi<br>ToNI*<br>ToL*
14	2020	Spain	5	5.55 (0.33)	0.4 (10)	25	Child-ANT<br>Stroop<br>Corsi<br>ToNI*<br>ToL*

(cont. in next page)

cont. Table S1

15	2021	Spain	4	4.35 (0.29)	0.60 (14)	23	Child-ANT<br>Stroop<br>Corsi<br>ToNI<br>ToL*
16	2021	Spain	4	4.34 (0.30)	0.62 (13)	21	Child-ANT<br>Stroop<br>Corsi<br>ToNI<br>ToL*
17	2021	Spain	4	4.32 (0.29)	0.47 (9)	19	Child-ANT<br>Stroop<br>Corsi<br>ToNI<br>ToL*
18	2021	Spain	4	4.25 (0.24)	0.5 (10)	20	Child-ANT<br>Stroop<br>Corsi<br>ToNI<br>ToL*
19	2021	Argentina	7	7.9 (0.41)	0.35 (7)	20	Child-ANT<br>Stroop<br>Corsi<br>ToNI
20	2021	Argentina	8	8.79 (0.48)	0.65 (11)	17	Child-ANT<br>Stroop<br>Corsi<br>ToNI
21	2022	Argentina	7	7.40 (0.30)	0.3 (6)	20	Child-ANT<br>Stroop<br>Corsi<br>ToNI
22	2023	Spain	4	4.56 (0.29)	(25)	42	Child-ANT<br>Stroop<br>Corsi<br>ToNI<br>ToL
23	2023	Spain	4	Missing data	(11)	23	Child-ANT<br>Stroop<br>Corsi<br>ToNI<br>ToL
24	2023	Spain	4	4.64 (0.28)	(20)	49	Child-ANT<br>Stroop<br>Corsi<br>ToNI<br>ToL

* Missing RT data

** RT data incomplete

brain sciences

MDPI

Section 2.2.1. Description of the time-unconstrained tasks

ToNI – 4 task

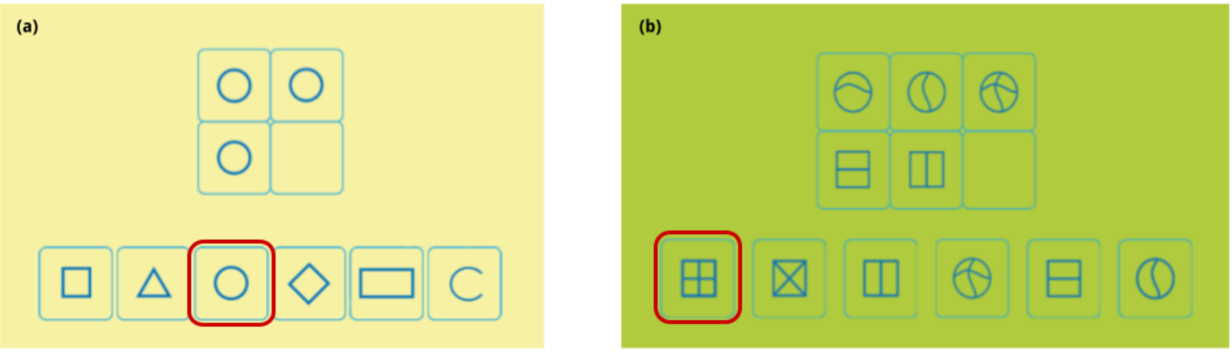

Figure S1. Two example trials from the adaptation of the ToNI task with different cognitive demands. In both examples the stimulus set is shown above, while the set of options is below. (a) The player must recognize that the relation between the figures in the stimulus set is that they are all the same geometric shape (circle) and choose, from the set of options, the missing figure that follows that logic (correct response is highlighted in red). (b) In this harder trial, the player must recognize that the bottom 3 drawings in the stimulus set must follow the same logic as the top 3: having the same geometric shape and following the same sequence of lines. To fulfil this, the missing drawing must be a square and have two perpendicular lines (correct response is highlighted in red).

Tower of London task

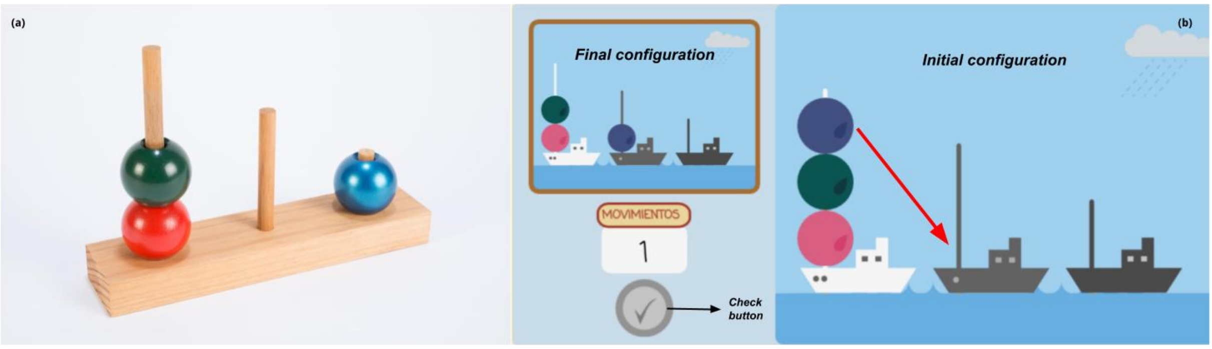

Figure S2. Original manual display and computerized version of the Tower of London task. (a) Wooden artefact used for the original version of this assessment. (b) Detailed screenshot of a trial from the computerized gamified version of the test. On the screen, the final configuration to be achieved is observed on the upper left, while the initial configuration that needs to be modified to reach the final configuration appears on the right. The check button that the player must press when they finished the trial is on the bottom left, indicated with an arrow. The desired number of movements is indicated below the final configuration (moving only one ball is sufficient to solve this trial). The figure also shows the different lengths of the rods, which can hold different number of balls: the shortest one, only one ball, the middle, two, and the longest one holds all three.

brain sciences

Section 2.2.2. Description of the time-constrained tasks

Child Attentional Networks Task

The Child Attentional Network Test (Child-ANT), an adaptation of its adult counterpart by Rueda and colleagues [1], was developed to evaluate three fundamental aspects of attentional processing in children, typically up to the age of around ten y.o- These aspects include alertness, orientation, and executive control. The task requires children to determine whether a central animal is pointing to the right or to the left and, then, signify their choice by pressing the corresponding arrow on the screen (highlighted in red in Figure S3(a)). Each trial in this test follows a specific sequence, as depicted in Figure 4 (main manuscript).

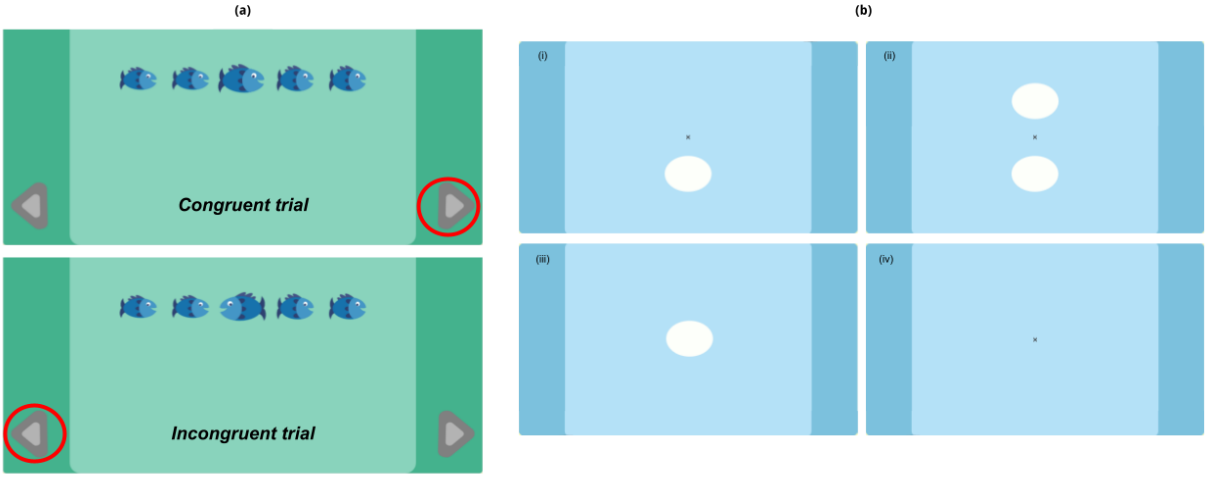

Figure S3. Captures of the Child-ANT task version. (a) Screenshots of test trials. Above, a congruent trial, in which all animals look in the same direction. Below, an incongruent trial in which the central animal looks in the opposite direction to the flanking animals. The correct response in both trials is the button marked in red. (b) Screenshots of the visual cues that appear before the target. (i) Spatial cue (in this case, below), (ii) Double cue, (iii) Central cue, (iv) Trial without a cue.

In 75% of the trials conducted, the screen presents one of three spatial cues 150 msec before displaying the animals. These cues fall into three categories: a) cues positioned in the same place where the target would appear, either above or below the center of the screen ('spatial'), b) cues positioned both above and below the center of the screen ('double'), or c) cues positioned at the center of the screen ('central') (referred to as (i), (ii), and (iii) respectively in Figure S3(b)). The remaining 25% of trials do not include any cues (referred to as (iv)). It's important to note that these cues are never explicitly explained to the children. The spatial cue (i) is strategically placed where the central animal will appear, effectively directing the player's focus to that particular location. This cue provides clear guidance regarding where the player should concentrate his/her attention. In contrast, the double cue (ii) serves as an alert that a stimulus is imminent, but doesn't offer any specific information about its exact screen position. The purpose of these trials is to investigate whether the response differs when participants are alerted about an impending stimulus compared to trials without cues. This component helps assess the alerting attention network. The central cue (iii), much like the double cue, does not indicate the stimulus's position. Instead, it functions as a 'useless' spatial cue. Comparing trials with central cue to trials with a spatial cue that effectively indicates the animal's position allows to estimate the orienting attention network. This helps determine if the presence of a spatial cue, even if it doesn't provide relevant information, affects performance.

brain sciences

MDPI

Heart-Flower Stroop task

This adapted Stroop task (the Heart-Flower Stroop), developed by Davidson and collaborators [2], serves as an assessment tool for measuring inhibitory control and cognitive flexibility in children. In this task, participants are required to press one of two buttons based on the appearance and position of visual stimuli presented in the screen. An example of a trial is depicted in Figure S4..

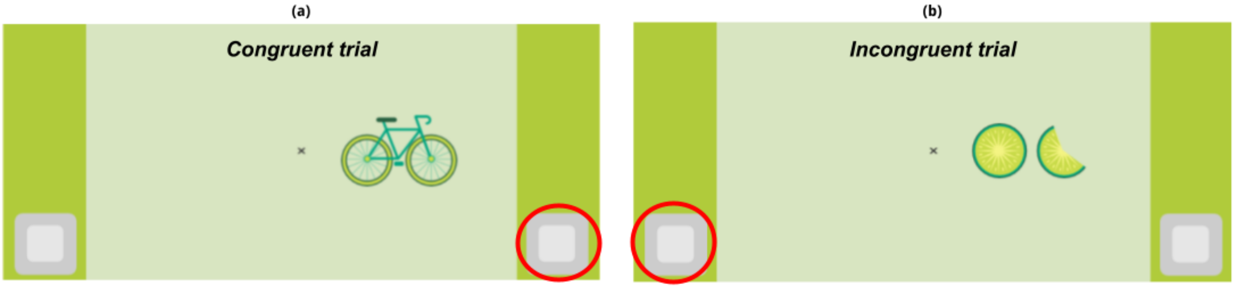

Figure S4. A summary of the Heart-Flower Stroop task used along the interventions. (a) congruent stimuli; (b) incongruent stimuli. In both cases, the correct answer is shown in red.

Section 2.4.1. *Data cleansing process and descriptive statistics*

We performed a correlation analysis with all the outcome variables resulting from the interventions. For the time-constrained tasks, however, we decided to only include performance on the incongruent trials. In the full analysis for both tasks (child-ANT and Heart-Flower Stroop) we do compare congruent to incongruent trials, but for this descriptive plot comparing both trial types within each evaluation is out of the scope.

Section 3.1. *Descriptive statistics*

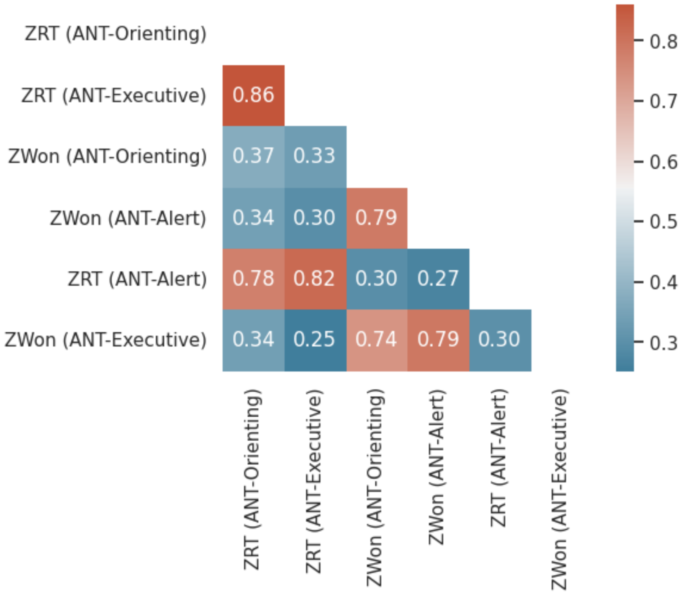

Figure S5. Correlation between outcome measures related to the three attentional networks measured with the child-ANT task.

brain sciences

MDPI

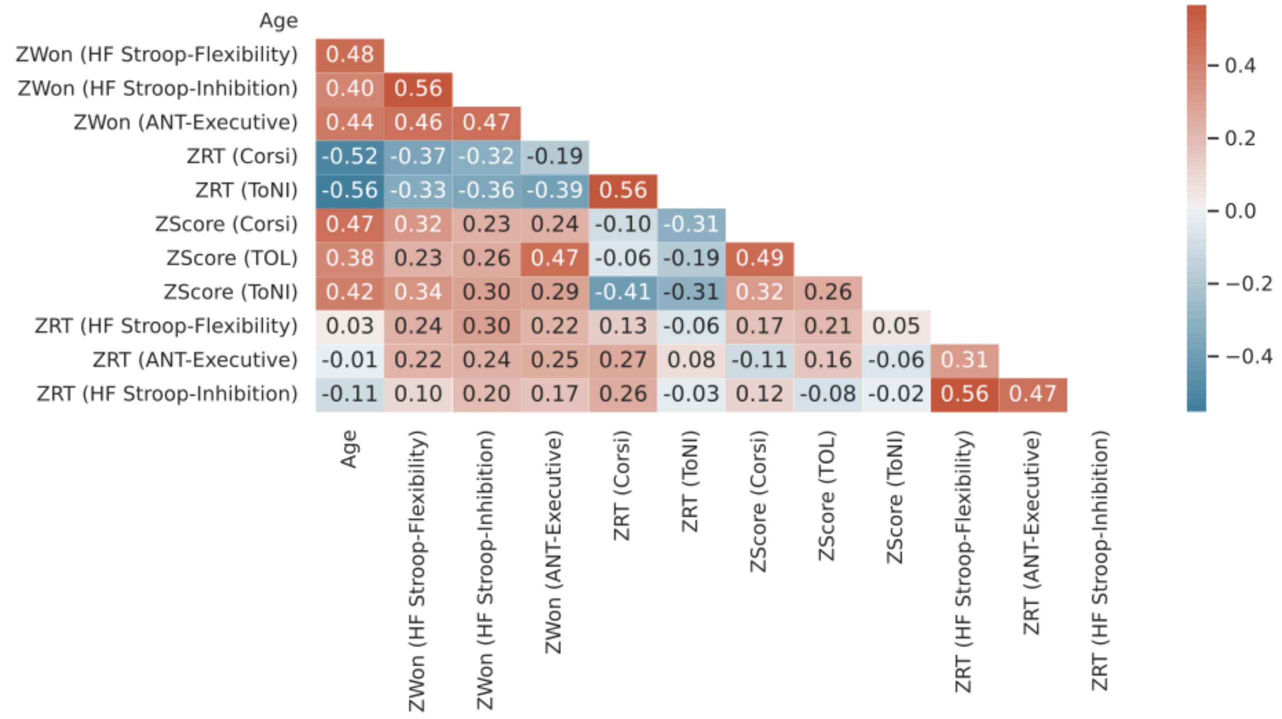

Figure S6. Heatmap depicting zero-order correlation between all outcome measurements (except for the Orientation and Alerting networks). Order is based on the correlation to the Age variable (cont).

Table S2. Bivariate correlations between all outcome variables and Age. Last column represents FDR corrected p-values.

Variable 1	Variable 2	Correlation	p-value	P-value (corrected)
Age	ZScore (Corsi)	0.47	1.441E-24	**2.377E-23**
Age	ZRT (Corsi)	-0.52	2.787E-30	**1.840E-28**
Age	ZWon (HF Stroop-Inhibition)	0.4	4.905E-14	**3.237E-13**
Age	ZRT (HF Stroop-Inhibition)	-0.11	4.836E-02	6.138E-02
Age	ZWon (HF Stroop-Flexibility)	0.48	4.360E-18	**3.597E-17**
Age	ZRT (HF Stroop-Flexibility)	0.03	6.437E-01	6.744E-01
Age	ZWon (ANT-Executive)	0.44	9.985E-13	**5.492E-12**
Age	ZRT (ANT-Executive)	-0.01	8.248E-01	8.248E-01
Age	ZScore (ToNI)	0.42	4.804E-19	**4.530E-18**
Age	ZRT (ToNI)	-0.56	1.628E-19	**2.149E-18**
Age	ZScore (TOL)	0.38	8.206E-09	**2.850E-08**
ZScore (Corsi)	ZRT (Corsi)	-0.1	2.094E-02	**2.880E-02**
ZScore (Corsi)	ZWon (HF Stroop-Inhibition)	0.23	2.566E-05	**4.981E-05**
ZScore (Corsi)	ZRT (HF Stroop-Inhibition)	0.12	3.983E-02	5.154E-02
ZScore (Corsi)	ZWon (HF Stroop-Flexibility)	0.32	3.350E-08	**1.005E-07**
ZScore (Corsi)	ZRT (HF Stroop-Flexibility)	0.17	4.198E-03	**6.156E-03**
(cont. in next page)				

brain sciences

(cont. Table S2)				
ZScore (Corsi)	ZWon (ANT-Executive)	0.24	7.893E-05	**1.408E-04**
ZScore (Corsi)	ZRT (ANT-Executive)	-0.11	6.570E-02	8.182E-02
ZScore (Corsi)	ZScore (ToNI)	0.32	1.830E-10	**7.548E-10**
ZScore (Corsi)	ZRT (ToNI)	-0.31	2.237E-06	**5.469E-06**
ZScore (Corsi)	ZScore (TOL)	0.49	3.233E-13	**1.940E-12**
ZRT (Corsi)	ZWon (HF Stroop-Inhibition)	-0.32	4.923E-09	**1.805E-08**
ZRT (Corsi)	ZRT (HF Stroop-Inhibition)	0.26	3.882E-06	**9.151E-06**
ZRT (Corsi)	ZWon (HF Stroop-Flexibility)	-0.37	1.335E-10	**5.872E-10**
ZRT (Corsi)	ZRT (HF Stroop-Flexibility)	0.13	2.851E-02	**3.841E-02**
ZRT (Corsi)	ZWon (ANT-Executive)	-0.19	1.812E-03	**2.917E-03**
ZRT (Corsi)	ZRT (ANT-Executive)	0.27	8.107E-06	**1.687E-05**
ZRT (Corsi)	ZScore (ToNI)	-0.41	1.055E-16	**7.739E-16**
ZRT (Corsi)	ZRT (ToNI)	0.56	4.199E-19	**4.530E-18**
ZRT (Corsi)	ZScore (TOL)	-0.06	4.065E-01	4.471E-01
ZWon (HF Stroop-Inhibition)	ZRT (HF Stroop-Inhibition)	0.2	1.351E-04	**2.346E-04**
ZWon (HF Stroop-Inhibition)	ZWon (HF Stroop-Flexibility)	0.56	4.499E-25	**9.897E-24**
ZWon (HF Stroop-Inhibition)	ZRT (HF Stroop-Flexibility)	0.3	2.548E-07	**7.008E-07**
ZWon (HF Stroop-Inhibition)	ZWon (ANT-Executive)	0.47	2.595E-12	**1.318E-11**
ZWon (HF Stroop-Inhibition)	ZRT (ANT-Executive)	0.24	8.056E-04	**1.329E-03**
ZWon (HF Stroop-Inhibition)	ZScore (ToNI)	0.3	1.573E-07	**4.514E-07**
ZWon (HF Stroop-Inhibition)	ZRT (ToNI)	-0.36	5.551E-07	**1.466E-06**
ZWon (HF Stroop-Inhibition)	ZScore (TOL)	0.26	3.593E-03	**5.646E-03**
ZRT (HF Stroop-Inhibition)	ZWon (HF Stroop-Flexibility)	0.1	9.577E-02	1.149E-01
ZRT (HF Stroop-Inhibition)	ZRT (HF Stroop-Flexibility)	0.56	3.540E-25	**9.897E-24**
ZRT (HF Stroop-Inhibition)	ZWon (ANT-Executive)	0.17	1.523E-02	**2.185E-02**
ZRT (HF Stroop-Inhibition)	ZRT (ANT-Executive)	0.47	4.157E-12	**1.960E-11**
ZRT (HF Stroop-Inhibition)	ZScore (ToNI)	-0.02	7.220E-01	7.331E-01
ZRT (HF Stroop-Inhibition)	ZRT (ToNI)	-0.03	7.110E-01	7.331E-01
ZRT (HF Stroop-Inhibition)	ZScore (TOL)	-0.08	3.761E-01	4.207E-01
ZWon (HF Stroop-Flexibility)	ZRT (HF Stroop-Flexibility)	0.24	1.876E-05	**3.751E-05**
ZWon (HF Stroop-Flexibility)	ZWon (ANT-Executive)	0.46	4.943E-10	**1.919E-09**
ZWon (HF Stroop-Flexibility)	ZRT (ANT-Executive)	0.22	3.797E-03	**5.828E-03**
ZWon (HF Stroop-Flexibility)	ZScore (ToNI)	0.34	2.882E-08	**9.058E-08**
ZWon (HF Stroop-Flexibility)	ZRT (ToNI)	-0.33	3.066E-05	**5.782E-05**
ZWon (HF Stroop-Flexibility)	ZScore (TOL)	0.23	1.564E-02	**2.196E-02**
(cont. in next page)				

brain sciences

(cont. Table S2)				
ZRT (HF Stroop-Flexibility)	ZWon (ANT-Executive)	0.22	4.018E-03	**6.026E-03**
ZRT (HF Stroop-Flexibility)	ZRT (ANT-Executive)	0.31	4.059E-05	**7.442E-05**
ZRT (HF Stroop-Flexibility)	ZScore (ToNI)	0.05	4.574E-01	4.949E-01
ZRT (HF Stroop-Flexibility)	ZRT (ToNI)	-0.06	4.905E-01	5.222E-01
ZRT (HF Stroop-Flexibility)	ZScore (TOL)	0.21	3.237E-02	**4.273E-02**
ZWon (ANT-Executive)	ZRT (ANT-Executive)	0.25	8.177E-06	**1.687E-05**
ZWon (ANT-Executive)	ZScore (ToNI)	0.29	5.470E-06	**1.245E-05**
ZWon (ANT-Executive)	ZRT (ToNI)	-0.39	1.789E-08	**5.905E-08**
ZWon (ANT-Executive)	ZScore (TOL)	0.47	7.979E-06	**1.687E-05**
ZRT (ANT-Executive)	ZScore (ToNI)	-0.06	3.463E-01	3.941E-01
ZRT (ANT-Executive)	ZRT (ToNI)	0.08	2.759E-01	3.195E-01
ZRT (ANT-Executive)	ZScore (TOL)	0.16	1.429E-01	1.684E-01
ZScore (ToNI)	ZRT (ToNI)	-0.31	1.240E-06	**3.147E-06**
ZScore (ToNI)	ZScore (TOL)	0.26	1.929E-04	**3.264E-04**
ZRT (ToNI)	ZScore (TOL)	-0.19	9.429E-02	1.149E-01

Table S3. Bivariate correlations between all outcome variables controlling for age. In bold, FDR corrected significant p-values.

Variable 1	Variable 2	Partial Correlation	p-value	P-value (corrected)
ZScore (Corsi)	ZRT (Corsi)	0.05	3.092E-01	4.360E-01
ZScore (Corsi)	ZWon (HF Stroop-Inhibition)	0.06	3.437E-01	4.501E-01
ZScore (Corsi)	ZRT (HF Stroop-Inhibition)	0.13	2.308E-02	**5.290E-02**
ZScore (Corsi)	ZWon (HF Stroop-Flexibility)	0.03	5.826E-01	6.817E-01
ZScore (Corsi)	ZRT (HF Stroop-Flexibility)	0.09	1.591E-01	2.747E-01
ZScore (Corsi)	ZWon (ANT-Executive)	0.18	7.839E-03	**2.536E-02**
ZScore (Corsi)	ZRT (ANT-Executive)	0.05	4.733E-01	5.785E-01
ZScore (Corsi)	ZScore (ToNI)	0.18	7.105E-04	**3.329E-03**
ZScore (Corsi)	ZRT (ToNI)	0	1.000E+00	1.000E+00
ZScore (Corsi)	ZScore (TOL)	0.35	7.809E-07	**8.590E-06**
ZRT (Corsi)	ZWon (HF Stroop-Inhibition)	-0.16	6.080E-03	**2.090E-02**
ZRT (Corsi)	ZRT (HF Stroop-Inhibition)	0.31	9.204E-08	**1.265E-06**
ZRT (Corsi)	ZWon (HF Stroop-Flexibility)	-0.09	1.484E-01	2.747E-01
ZRT (Corsi)	ZRT (HF Stroop-Flexibility)	0.26	2.095E-05	**1.646E-04**
ZRT (Corsi)	ZWon (ANT-Executive)	0.07	3.222E-01	4.430E-01
ZRT (Corsi)	ZRT (ANT-Executive)	0.17	1.389E-02	**3.820E-02**
(cont. in next page)				

(cont. Table S3)				
ZRT (Corsi)	ZScore (ToNI)	-0.18	7.896E-04	**3.341E-03**
ZRT (Corsi)	ZRT (ToNI)	0.32	3.972E-06	**3.641E-05**
ZRT (Corsi)	ZScore (TOL)	-0.03	7.163E-01	7.577E-01
ZWon (HF Stroop-Inhibition)	ZRT (HF Stroop-Inhibition)	0.16	3.287E-03	**1.205E-02**
ZWon (HF Stroop-Inhibition)	ZWon (HF Stroop-Flexibility)	0.45	8.281E-15	**2.277E-13**
ZWon (HF Stroop-Inhibition)	ZRT (HF Stroop-Flexibility)	0.23	1.462E-04	**8.040E-04**
ZWon (HF Stroop-Inhibition)	ZWon (ANT-Executive)	0.29	5.726E-05	**3.936E-04**
ZWon (HF Stroop-Inhibition)	ZRT (ANT-Executive)	0.19	1.197E-02	**3.657E-02**
ZWon (HF Stroop-Inhibition)	ZScore (ToNI)	0.15	1.310E-02	**3.793E-02**
ZWon (HF Stroop-Inhibition)	ZRT (ToNI)	-0.11	1.529E-01	2.747E-01
ZWon (HF Stroop-Inhibition)	ZScore (TOL)	0.11	2.479E-01	3.752E-01
ZRT (HF Stroop-Inhibition)	ZWon (HF Stroop-Flexibility)	0.05	4.230E-01	5.288E-01
ZRT (HF Stroop-Inhibition)	ZRT (HF Stroop-Flexibility)	0.57	5.643E-24	**3.104E-22**
ZRT (HF Stroop-Inhibition)	ZWon (ANT-Executive)	0.09	2.129E-01	3.411E-01
ZRT (HF Stroop-Inhibition)	ZRT (ANT-Executive)	0.44	3.789E-10	**6.947E-09**
ZRT (HF Stroop-Inhibition)	ZScore (ToNI)	0.07	2.170E-01	3.411E-01
ZRT (HF Stroop-Inhibition)	ZRT (ToNI)	0.05	4.915E-01	5.877E-01
ZRT (HF Stroop-Inhibition)	ZScore (TOL)	-0.03	7.150E-01	7.577E-01
ZWon (HF Stroop-Flexibility)	ZRT (HF Stroop-Flexibility)	0.14	1.923E-02	**4.924E-02**
ZWon (HF Stroop-Flexibility)	ZWon (ANT-Executive)	0.27	7.262E-04	**3.329E-03**
ZWon (HF Stroop-Flexibility)	ZRT (ANT-Executive)	0.19	1.969E-02	**4.924E-02**
ZWon (HF Stroop-Flexibility)	ZScore (ToNI)	0.09	1.598E-01	2.747E-01
ZWon (HF Stroop-Flexibility)	ZRT (ToNI)	-0.04	6.163E-01	7.062E-01
ZWon (HF Stroop-Flexibility)	ZScore (TOL)	-0.01	9.535E-01	9.711E-01
ZRT (HF Stroop-Flexibility)	ZWon (ANT-Executive)	0.09	2.739E-01	3.965E-01
ZRT (HF Stroop-Flexibility)	ZRT (ANT-Executive)	0.26	1.297E-03	**5.097E-03**
ZRT (HF Stroop-Flexibility)	ZScore (ToNI)	0.03	6.681E-01	7.349E-01
ZRT (HF Stroop-Flexibility)	ZRT (ToNI)	0.08	3.349E-01	4.493E-01
ZRT (HF Stroop-Flexibility)	ZScore (TOL)	0.14	1.720E-01	2.867E-01
ZWon (ANT-Executive)	ZRT (ANT-Executive)	0.15	2.244E-02	5.290E-02
ZWon (ANT-Executive)	ZScore (ToNI)	0.11	1.001E-01	2.039E-01
ZWon (ANT-Executive)	ZRT (ToNI)	-0.13	7.982E-02	1.688E-01
ZWon (ANT-Executive)	ZScore (TOL)	0.42	1.214E-04	**7.421E-04**
ZRT (ANT-Executive)	ZScore (ToNI)	0.03	6.366E-01	7.145E-01
ZRT (ANT-Executive)	ZRT (ToNI)	0.11	1.296E-01	2.545E-01
(cont. in next page)				

brain sciences

(cont. Table S3)

ZRT (ANT-Executive)	ZScore (TOL)	0.13	2.524E-01	3.752E-01
ZScore (ToNI)	ZRT (ToNI)	-0.06	3.760E-01	4.800E-01
ZScore (ToNI)	ZScore (TOL)	0.14	4.629E-02	1.018E-01
ZRT (ToNI)	ZScore (TOL)	-0.02	8.694E-01	9.022E-01

Section 3.2. *Time-unconstrained tasks*

3.2.1. *Corsi task*

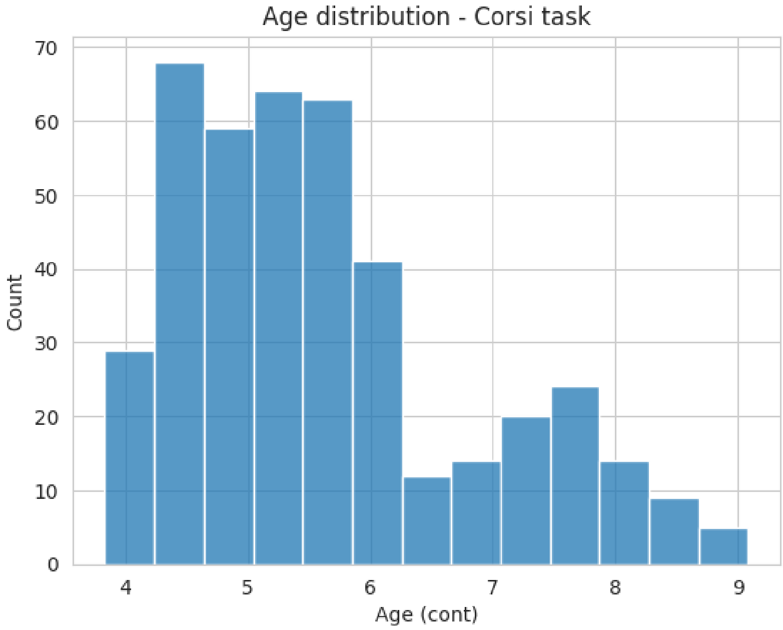

Figure S7. Age distribution for the participants of the Corsi task included in the analysis.

Table S4. Parameters for the linear mixed model with working memory Score (log) as the outcome and Age (cont) as predictor.

	**Working memory Score (log)**					
Predictors	Coefficient	Std. Error	z	P>|z|	[0.025	0.975]
(Intercept)	-2.129	0.724	-2.941	**0.003**	-3.548	-0.710
Age	2.669	0.416	6.417	**<0.001**	1.854	3.484
Group Var	0.178	0.080				
Observations	422					
Groups	23					
RMSE	0.975					
Marginal R^2	0.294					
Conditional R^2	0.423					

Table S5. Full model output for Score and RT (log) response variables in the Corsi Block-Tapping task. In bold the selected models and the best metric in each case.

Models	Comparison	metric	linear	logarithmic
**Score**				
i. log(Score) ~ 1 + (1∣Subset)	i. vs ii.	*LR: 32.55, p-value:1.16e-8*		
ii. log(Score) ~ Age + (1∣Subset)	ii. vs iii	metric	linear	logarithmic
iii. log(Score) ~ log(Age) + (1∣Subset)		RMSE	**0.970**	0.975
		R^2 (marginal)	**0.298**	0.294
		R^2 (conditional)	0.407	**0.423**
**RT**				
For difficulty level 1:	i. vs ii.	*LR: 14.49, p-value:1.41e-4*		
i. log(RT) ~ 1 + (1∣Subset)	ii. vs iii	metric	linear	logarithmic
ii. log(RT) ~ Age + (1∣Subset)		- RMSE	0.433	**0.423**
iii. log(RT) ~ log(Age) + (1∣Subset)		R^2 (marginal)	0.391	**0.397**
		R^2 (conditional)	**0.652**	0.651
For difficulty level 2:	i. vs ii.	*LR: 12.78, p-value:3.50e-4*		
i. log(RT) ~ 1 + (1∣Subset)	ii. vs iii	metric	linear	logarithmic
ii. log(RT) ~ Age + (1∣Subset)		- RMSE	0.398	**0.387**
iii. log(RT) ~ log(Age) + (1∣Subset)		R^2 (marginal)	0.400	**0.408**
		R^2 (conditional)	**0.671**	0.670
For difficulty level 3:	i. vs ii.	*LR: 8.21, p-value:4.19e-3*		
i. log(RT) ~ 1 + (1∣Subset)	ii. vs iii	metric	linear	logarithmic
ii. log(RT) ~ Age + (1∣Subset)		- RMSE	0.433	**0.423**
iii. log(RT) ~ log(Age) + (1∣Subset)		R^2 (marginal)	0.390	**0.397**
		R^2 (conditional)	**0.652**	0.651

brain sciences

Table S6.(a) Parameters for the linear mixed model with working memory RT (only stimulus amount = 1) as the outcome and Age (cont) as predictors. **(b)** Parameters for the linear mixed model with working memory RT (only stimulus amount = 2) as the outcome and Age (cont) as predictors. **(c)** Parameters for the logarithmic mixed model with working memory RT (only stimulus amount = 3) as the outcome and Age (cont) as predictors.

**(a)**	**working memory RT (log) – Stimulus amount = 1**					
Predictors	Coefficient	Std. Error	z	P>|z|	[0.025	0.975]
(Intercept)	3.665	0.355	10.328	<0.001	4.360	4.273
Age (log)	-0.898	0.202	-4.438	<0.001	-0.501	-0.475
Group Var	0.076	0.079				
Observations	422					
Groups	23					
RMSE	0.423					
Marginal R^2	0.397					
Conditional R^2	0.651					

**(b)**	**Working memory RT (log) – Stimulus amount = 2**					
Predictors	Coefficient	Std. Error	z	P>|z|	[0.025	0.975]
(Intercept)	3.828	0.360	10.644	<0.001	3.123	4.532
Age (log)	-0.879	0.204	-4.306	<0.001	-1.279	-0.479
Group Var	0.068	0.082				
Observations	326					
Groups	23					
RMSE	0.387					
Marginal R^2	0.408					
Conditional R^2	0.670					

**(c)**	**Working memory RT (log) – Stimulus amount = 3**					
Predictors	Coefficient	Std. Error	z	P>|z|	[0.025	0.975]
(Intercept)	3.737	0.387	9.663	<0.001	2.979	4.495
Age (log)	-0.701	0.219	-3.205	0.001	-1.129	-0.272
Group Var	0.056	0.088				
Observations	187					
Groups	23					
RMSE	0.327					
Marginal R^2	0.324					
Conditional R^2	0.651					

Table S7. Mixed regression results for the RT outcome variable in the Corsi Block-Tapping task.

	**Working memory RT – Difficulty effect**					
Predictors	*Coefficient*	*Std. Error*	*z*	*P>|z|*	*[0.025*	*0.975]*
(Intercept)	2.715	0.159	17.121	**<0.0001**	2.404	3.026
Age	-0.132	0.025	-5.191	**<0.0001**	-0.182	0.082
Stimulus amount	0.123	0.035	3.535	**<0.0001**	0.055	0.191
Age:Stimulus Amount	0.010	0.005	1.847	0.065	-0.001	0.020
Group Var	0.075	0.086				
Observations	1141					
Groups	24					
RMSE	0.370					
Marginal R^2	0.452					
Conditional R^2	0.730					

brain sciences MDPI

3.2.2. *ToNI task*

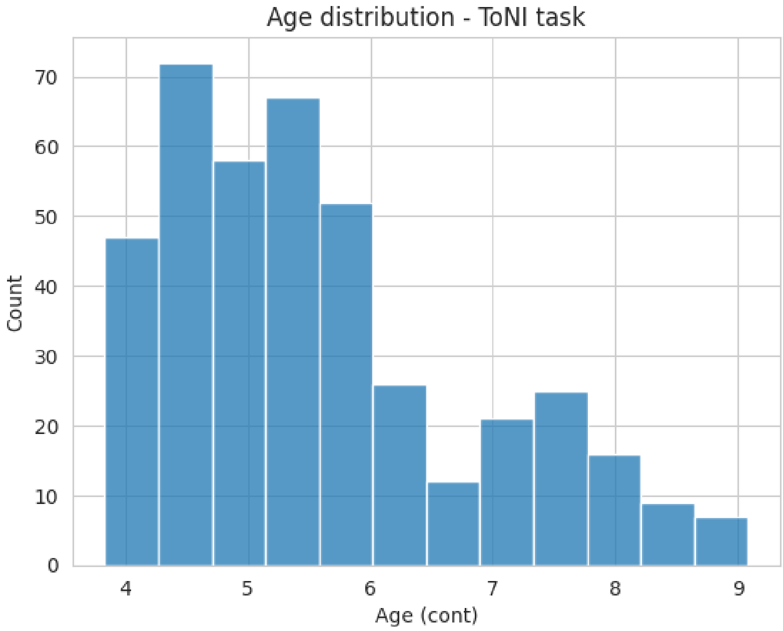

Figure S8. Age distribution for the participants of the ToNI task included in the analysis.

Table S8. Parameters for the logarithmic mixed model with relational Score as the outcome and Age (log) as predictor.

	**Relational Score (log)**					
Predictors	*Coefficient*	*Std. Error*	*z*	*P>|z|*	*[0.025*	*0.975]*
(Intercept)	1.257	0.0.378	3.322	**0.001**	0.515	1.999
Age (log)	1.494	0.219	6826	**<0.001**	1.065	1.923
Group Var	0.024	0.023				
Observations	412					
Groups	22					
RMSE	0.704					
Marginal R^2	0.188					
Conditional R^2	0.228					

Table S9. Parameters for the logarithmic mixed model with log relational RT as the outcome and Age (log) and Trial Number (log) as predictors.

	**Relational RT (log)**					
Predictors	*Coefficient*	*Std. Error*	*z*	*P>|z|*	*[0.025*	*0.975]*
(Intercept)	1.475	0.213	6.908	**<0.001**	1.056	1.893
Age (log)	-0.765	0.117	-6.546	**<0.001**	-0.995	-0.536
Trial Number (log)	0.724	0.036	199.924	**<0.001**	0.653	0.795
Group Var	0.012	0.015				
Observations	1418					
Groups	12					
RMSE	0.453					
Marginal R^2	0.305					
Conditional R^2	0.346					

Table S10. Full model output for Score and RT (log) response variables in the ToNI-4 task. In bold the selected models and the best metric in each case.

Score				
**Models**	**Comparison**			
	i. vs ii.	*LR: 30.32 , p-value: 3.67 e-8*		
i. log(Score) ~ 1 + (1 ǀ Subset) *ii. log(Score) ~ Age + (1 ǀ Subset)* ***iii. log(Score) ~ log(Age) + (1 ǀ Subset)***	ii. vs iii	metric	linear	logarithmic
		- RMSE	0.708	**0.704**
		R^2 (marginal)	0.182	**0.188**
		R^2 (conditional)	0.223	**0.227**

RT				
**Models**	**Comparison**			
i. log(RT) ~ 1 + (1 ǀ Subset) *ii. log(RT) ~ Trial Number + (1 ǀ Subset)* *iii. log(RT) ~ Age + (1 ǀ Subset)* *iv. log(RT) ~ Trial Number + Age + (1 ǀ Subset)* *v. log(RT) ~ Trial Number + Age + Trial Number:Age + (1 ǀ Subset)* ***vi. log(RT) ~ Trial Number + log(Age) + (1 ǀ Subset)***	i. vs ii.	*LR: 309.29, p-value:3.11e-69*		
	i vs iii.	*LR: 7.70, p-value: 5.5e-3*		
	ii. vs iii	metric	linear	logarithmic
		- RMSE	**0.512**	0.514
		R^2 (marginal)	**0.246**	0.118
		R^2 (conditional)	**0.430**	0.165
	ii. vs iv.	*LR: 19.64, p-value: 9.33 e-6*		
	iv. vs v.	*LR: 1.45, p-value: 0.23*		
	v. vs vi	metric	linear	logarithmic
		- RMSE	0.459	**0.453**
		R^2 (marginal)	0.291	**0.305**
		R^2 (conditional)	0.327	**0.345**

3.2.3. *ToL task*

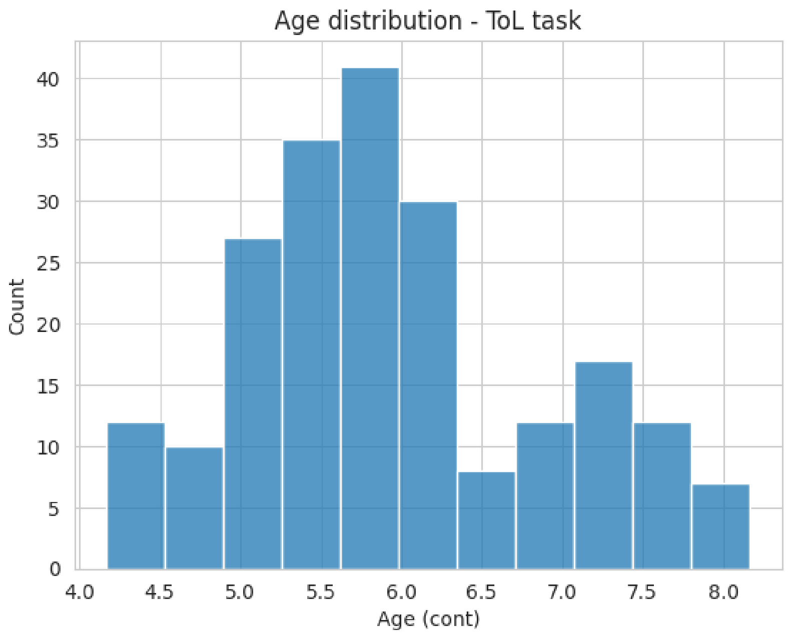

Figure S9. Age distribution for the participants of the ToL task included in the analysis

brain sciences

MDPI

Table S11. Parameters for the logarithmic mixed model with planning Score as the outcome and Age (log) as predictors.

			**Planning Score (log)**			
Predictors	Coefficient	Std. Error	z	P>|z|	[0.025	0.975]
(Intercept)	-2.488	1.544	-1.611	0.107	-5.514	0.539
Age (log)	2.887	0.863	3.346	**0.001**	1.196	4.578
Group Var	0.299	0.170				
Observations	211					
Groups	11					
RMSE	1.076					
Marginal R^2	0.224					
Conditional R^2	0.415					

Table S12. Full model output for Score variable in the ToL task.

**Score**				
	i. vs ii.		*LR: 10.03, p-value:1.5e-3*	
i. log(Score) ~ 1 + (1|Subset)		metric	linear	logarithmic
ii. log(Score) ~ Age + (1|Subset)	ii. vs iii	- RMSE	1.077	**1.076**
iii. log(Score) ~ log(Age) + (1|Subset)		R^2 (marginal)	0.224	**0.224**
		R^2 (conditional)	0.390	**0.415**

Section 3.3. *Time-constrained tasks*

3.3.1. Child-ANT task

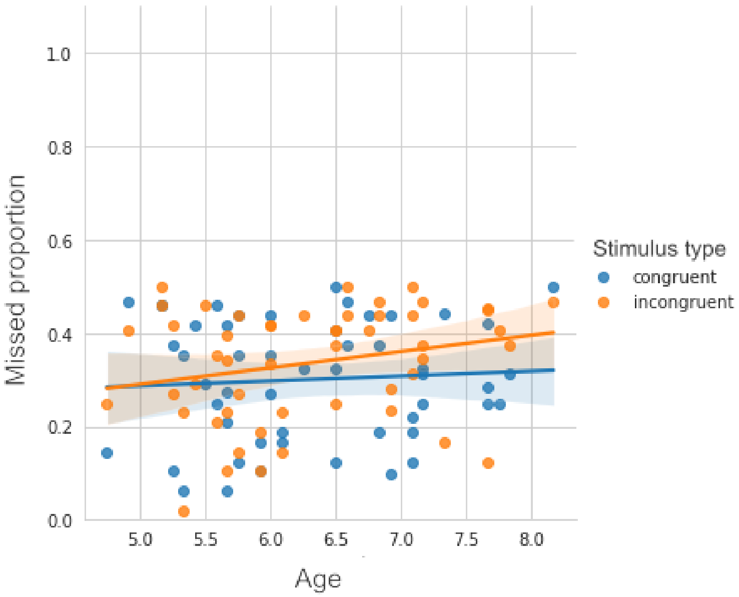

Figure S10. Proportion of missed trials in the original version of the Child-ANT task.

brain sciences

MDPI

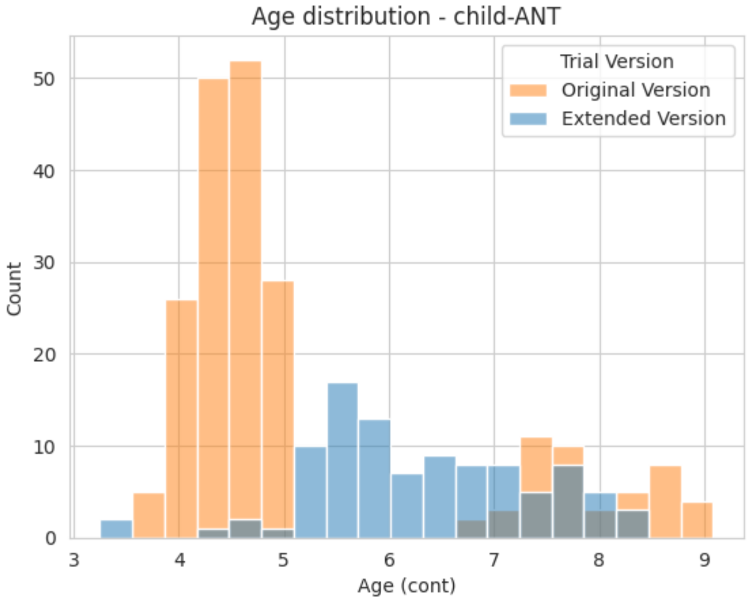

Figure S11. Age distribution for the original version (orange) and the extended version (blue) of the Child-ANT task

Table S13. Parameters for the logarithmic mixed model with ZWon as the outcome and Age (log) and Stimulus Type as predictors.

**ZWon – Original version**						
Predictors	Coefficient	Std. Error	z	P>|z|	[0.025	0.975]
(Intercept)	-3.177	0.872	-3.641	**<0.001**	-4.886	-1.467
Age (log)	1.909	0.456	4.103	**<0.001**	0.997	2.821
stimulus_type	-0.683	0.083	-8.186	**<0.001**	-0.847	-0.520
Group Var	0.030	0.039				
Observations	471					
Groups	7					
RMSE	0.911					
Marginal R^2	0.184					
Conditional R^2	0.213					

Table S14. Parameters for the null mixed model with ZRT as the outcome.

**ZRT– Original version**						
Predictors	Coefficient	Std. Error	z	P>|z|	[0.025	0.975]
(Intercept)	0.132	0.170	0.780	0.435	-0.200	0.465
Group Var	0.165	0.102				
Observations	471					
Groups	7					
RMSE	1.089					
Marginal R^2	0.116					
Conditional R^2	0.239					

brain sciences MDPI

Table S15. Parameters for the logarithmic mixed model with ZWon as the outcome and Age (log) and Stimulus Type as predictors.

ZWon – Extended version

Predictors	Coefficient	Std. Error	z	P>|z|	[0.025	0.975]
(Intercept)	-1.719	0.302	-5.687	**<0.001**	-2.312	-1.127
Age (log)	1..220	0.290	6.793	**<0.001**	0.868	1.572
Stimulus Type	-2.139	0.180	-7.371	**<0.001**	-2.707	-1.570
Age (log):Stimulus Type	0.971	0.176	5.502	**<0.001**	0.625	1.317
Group Var	0.015	0.012				
Observations	1522					
Groups	9					
RMSE	0.866					
Marginal R^2	0.264					
Conditional R^2	0.279					

Table S16. Parameters for the logarithmic mixed model with ZRT as the outcome and Age (log), Cue and Stimulus Type as predictors.

ZRT – Extended version

Predictors	Coefficient	Std. Error	z	P>|z|	[0.025	0.975]
(Intercept)	-1.177	0.322	-3.657	**<0.0001**	-1.808	-0.546
Stimulus Type[T.incongruent]	0.294	0.049	6.045	**<0.001**	0.199	0.390
cue[T.double]	0.005	0.069	0.072	0.943	-0.130	0.139
cue[T.no_cue]	0.117	0.069	1.697	0.090	-0.018	0.252
cue[T.spatial]	-0.014	0.069	-0.201	0.841	-0.149	0.121
Age (log)	0.611	0.187	3.260	**0.001**	0.244	0.978
Group Var	0.024	0.017				
Observations	1522					
Groups	9					
RMSE	0.957					
Marginal R^2	0.066					
Conditional R^2	0.090					

brain sciences

MDPI

Table S17. Full model output for Won and ZRT response variables in the original version of the child-ANT task.In bold the selected models and the best metric in each case.

ZWon

	Models
i.	*ZWon ~ 1 + (1 ǀ Subset)*
ii.	*ZWon ~ Age + (1 ǀ Subset)*
iii.	*ZWon ~ Cue + (1 ǀ Subset)*
iv.	*ZWon ~ Stimulus Type + (1 ǀ Subset)*
v.	*ZWon ~ Stimulus Type + Age + (1 ǀ Subset)*
vi.	*ZWon ~ Stimulus Type + Cue + (1 ǀ Subset)*
vii.	***ZWon ~ Stimulus Type + log(Age) + (1 ǀ Subset)***

Comparison	
i. vs ii.	*LR: 22.57, p-value: 2.02 e-6*
i. vs iii.	*LR: 2.80, p-value: 0.094*
i. vs iv.	*LR: 64.55, p-value: 9.40*

ii. vs iii vs iv. metric	null	age	cue	stim
- RMSE	1.005	0.978	1.002	**0.940**
R^2 (marginal)	0.023	0.067	0.029	**0.146**
R^2 (conditional)	0.059	0.096	0.065	**0.180**

Comparison	
ii. vs v.	*LR: 21.04, p-value: 4.94 e-6*
ii. vs vi.	*LR: 1.96, p-value: 0.16*

vi. vs vii metric	linear	logarithmic
- RMSE	0.938	**0.911**
R^2 (marginal)	0.149	**0.184**
R^2 (conditional)	0.184	**0.213**

ZRT

	Models	Comparison	
i.	***ZRT ~ 1 + (1 ǀ Subset)***	i. vs ii.	*LR: 0.16 , p-value: 0.68*
ii.	*ZRT ~ Age + (1 ǀ Subset)*	i vs iii.	*LR: 0.30 , p-value: 0.58*
iii.	*ZRT ~ Cue + (1 ǀ Subset)*	i. vs iv.	*LR: 1.09 , p-value: 0.29*
iv.	*ZRT ~ Stimulus Type + (1 ǀ Subset)*		

brain sciences

MDPI

Table S18. Full model output for Won and ZRT response variables in the extended version of the child-ANT task.In bold the selected models and the best metric in each case.

<table>
<tr><th colspan="7">ZWon</th></tr>
<tr><th>Models</th><th colspan="6">Comparison</th></tr>
<tr><td rowspan="15">i. ZWon ~ 1 + (1|Subset)
ii. ZWon ~ Age + (1|Subset)
iii. ZWon ~ Cue + (1|Subset)
iv. ZWon ~ Stimulus Type + (1|Subset)
v. ZWon ~ Stimulus Type + Age + (1|Subset)
vi. ZWon ~ Stimulus Type + Cue + (1|Subset)
vii. ZWon~ Stimulus Type + Age + Stimulus_type:Age + (1|Subset)
viii. ZWon ~ Stimulus Type + log(Age) + Stimulus Type:log(Age) + (1|Subset)</td><td>i. vs ii.</td><td colspan="5">LR: 40.22, p-value: 2.27 e-10</td></tr>
<tr><td>i. vs iii.</td><td colspan="5">LR: 1.99, p-value: 0.15</td></tr>
<tr><td>i. vs iv.</td><td colspan="5">LR: 148.55, p-value: 3.59 e-34</td></tr>
<tr><td rowspan="4">ii. vs iii vs iv.</td><td>metric</td><td>null</td><td>age</td><td>cue</td><td>stim</td></tr>
<tr><td>- RMSE</td><td>1.009</td><td>0.921</td><td>1.009</td><td>0.971</td></tr>
<tr><td>R^2 (marginal)</td><td>0.131</td><td>0.169</td><td>0.132</td><td>0.195</td></tr>
<tr><td>R^2 (conditional)</td><td>0.312</td><td>0.180</td><td>0.312</td><td>0.378</td></tr>
<tr><td>iv. vs v.</td><td colspan="5">LR: 21.04, p-value: 4.94 e-6</td></tr>
<tr><td>iv. vs vi.</td><td colspan="5">LR: 1.96, p-value: 0.16</td></tr>
<tr><td>v. vs vii.</td><td colspan="5">LR: 30.13, p-value:4.04e-8</td></tr>
<tr><td rowspan="4">vii. vs viii</td><td colspan="2">metric</td><td>linear</td><td colspan="2">logarithmic</td></tr>
<tr><td colspan="2">- RMSE</td><td>0.869</td><td colspan="2">0.866</td></tr>
<tr><td colspan="2">R^2 (marginal)</td><td>0.261</td><td colspan="2">0.264</td></tr>
<tr><td colspan="2">R^2 (conditional)</td><td>0.274</td><td colspan="2">0.279</td></tr>
<tr><td colspan="6"></td></tr>
<tr><th colspan="7">ZRT</th></tr>
<tr><th>Models</th><th colspan="6">Comparison</th></tr>
<tr><td rowspan="21">i. ZRT ~ 1 + (1|Subset)
ii. ZRT ~ Age + (1|Subset)
iii. ZRT ~ Cue + (1|Subset)
iv. ZRT ~ Stimulus Type + (1|Subset)
v. ZRT ~ Stimulus Type + Age + (1|Subset)
vi. ZRT ~ Stimulus Type + Cue + (1|Subset)
vii. ZRT ~ Stimulus Type + Age + Cue + (1|Subset)
viii. ZRT ~ Stimulus Type + Age + Cue + Stimulus Type:Age + (1|Subset)
ix. ZRT ~ Stimulus Type + Age + Cue + Stimulus Type:Cue + (1|Subset)
x. ZRT ~ Stimulus Type + Age + Cue + Age:Cue + (1|Subset)
xi. ZRT ~ Stimulus Type + log(Age) + Cue + (1|Subset)</td><td>i. vs ii.</td><td colspan="5">LR: 8.07, p-value: 4.4 e-3</td></tr>
<tr><td>i. vs iii.</td><td colspan="5">LR: 4.76, p-value: 2.9 e-2</td></tr>
<tr><td>i. vs iv.</td><td colspan="5">LR: 36.30, p-value: 1.69 e-9</td></tr>
<tr><td rowspan="4">ii. vs iii vs iv.</td><td>metric</td><td>null</td><td>age</td><td>cue</td><td>stim</td></tr>
<tr><td>RMSE</td><td>0.984</td><td>0.969</td><td>0.982</td><td>0.972</td></tr>
<tr><td>R^2 (marginal)</td><td>0.037</td><td>0.039</td><td>0.039</td><td>0.059</td></tr>
<tr><td>R^2 (conditional)</td><td>0.087</td><td>0.059</td><td>0.089</td><td>0.107</td></tr>
<tr><td>ii. vs v.</td><td colspan="5">LR: 8.19, p-value: 4.2 e-3</td></tr>
<tr><td>ii. vs vi.</td><td colspan="5">LR: 4.58, p-value: 3.2e-2</td></tr>
<tr><td rowspan="4">v. vs vi.</td><td>metric</td><td>stim.</td><td>stim:age</td><td colspan="2">stim:cue</td></tr>
<tr><td>RMSE</td><td>0.972</td><td>0.582</td><td colspan="2">0.971</td></tr>
<tr><td>R^2 (marginal)</td><td>0.058</td><td>0.062</td><td colspan="2">0.061</td></tr>
<tr><td>R^2 (conditional)</td><td>0.107</td><td>0.080</td><td colspan="2">0.110</td></tr>
<tr><td>v. vs vii.</td><td colspan="5">LR: 4.58, p-value: 3.2e-2</td></tr>
<tr><td>vii. vs viii.</td><td colspan="5">LR: 0.05, p-value: 0.82</td></tr>
<tr><td>vii. vs ix.</td><td colspan="5">LR: 0.58, p-value: 0.44</td></tr>
<tr><td>vii. vs x.</td><td colspan="5">LR: 2.32, p-value: 0.13</td></tr>
<tr><td rowspan="4">vii. vs xi.</td><td colspan="2">metric</td><td>linear</td><td colspan="2">logarithmic</td></tr>
<tr><td colspan="2">RMSE</td><td>0.957</td><td colspan="2">0.957</td></tr>
<tr><td colspan="2">R^2 (marginal)</td><td>0.064</td><td colspan="2">0.065</td></tr>
<tr><td colspan="2">R^2 (conditional)</td><td>0.083</td><td colspan="2">0.090</td></tr>
</table>

brain sciences

MDPI

3.3.2. *The Heart-Flower Stroop task*

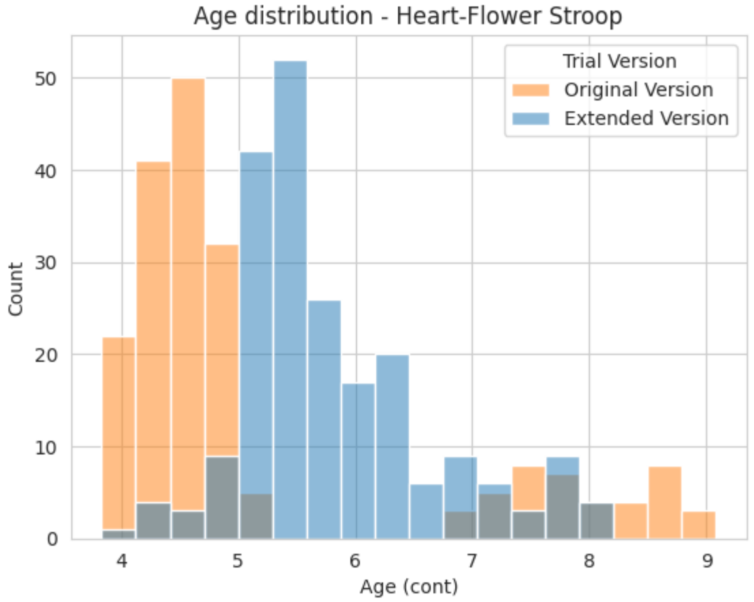

Figure S12. Age distribution for the original version (orange) and the extended version (blue) of the Stroop Flower-Heart task.

Table S19. Parameters for the linear mixed model with ZWon as the outcome and Block as predictor.

ZWon – Original version

Predictors	*Coefficient*	*Std. Error*	*z*	*P>|z|*	*[0.025*	*0.975]*
(Intercept)	0.832	0.118	7.061	**<0.0001**	0.601	1.063
block	-0.437	0.047	-9.215	**<0.0001**	-0.530	-0.344
Group Var	0.047	0.028				
Observations	541					
Groups	14					
RMSE	0.160					
Marginal R^2	0.179					
Conditional R^2	0.206					

Table S20. Parameters for the logarithmic mixed model with ZWon as the outcome and log Age (cont) and Block as predictors.

ZWon – Extended version

Predictors	*Coefficient*	*Std. Error*	*z*	*P>|z|*	*[0.025*	*0.975]*
(Intercept)	-2.306	0.401	-5.755	**<0.0001**	-3.091	-1.520
Age (log)	-0.324	0.044	-7.362	**<0.001**	-0.410	-0.237
block	1.834	0.233	7.874	**<0.0001**	1.377	2.290
Group Var	0.022	0.024				
Observations	540					
Groups	9					
RMSE	0.844					
Marginal R^2	0.303					
Conditional R^2	0.324					

brain sciences

MDPI

Table S21. Parameters for the logarithmic mixed model with RT (log) as the outcome and Age (cont) and Block as predictors.

RT (log) – Original version

Predictors	*Coefficient*	*Std. Error*	*z*	*P>|z|*	*[0.025*	*0.975]*
(Intercept)	2.511	0.884	2.839	**0.005**	0.778	4.244
block	0.216	0.049	4.420	**<0.0001**	0.120	0.312
Age (log)	-1.689	0.502	-3.366	**0.001**	-2.672	-0.706
Group Var	0.058	0.043				
Observations	541					
Groups	14					
RMSE	0.957					
Marginal R^2	0.132					
Conditional R^2	0.186					

Table S22. Parameters for the linear mixed model with ZRT as the outcome and Age (cont) and Block as predictors.

ZRT – Extended version

Predictors	*Coefficient*	*Std. Error*	*z*	*P>|z|*	*[0.025*	*0.975]*
(Intercept)	-1.371	0.291	-4.711	**<0.0001**	-1.942	-0.801
block	0.116	0.048	2.434	0.245	0.023	0.210
Age	0.222	0.048	4.658	**<0.0001**	0.129	0.315
Group Var	0.040	0.033				
Observations	540					
Groups	9					
RMSE	0.926					
Marginal R^2	0.161					
Conditional R^2	0.200					

Table S23. Full model output for Won and ZRT response variables in the original version of the Stroop task.In bold the selected models and the best metric in each case.

ZWon

Models	Comparison	
i. ZWon ~ 1 + (1|Subset)	i. vs ii.	*LR: 1.74, p-value: 0.187*
ii. ZWon ~ Age + (1|Subset)		
iii. ZWon ~ Block + (1|Subset)	i. vs iii.	*LR: 78.69, p-value: 7.27 e-19*

ZRT

Models	Comparison			
	i. vs ii.	*LR: 9.95, p-value: 1.60 e-2*		
i. ZRT ~ 1 + (1|Subset)	i vs iii.	*LR: 18.77, p-value: 1.47 e-5*		
ii. ZRT ~ Age + (1|Subset)	ii. vs iv.	*LR: 10.55, p-value: 1.16 e-2*		
iii. ZRT ~ Block + (1|Subset)	iv. vs v.	*LR: 0.37, p-value: 0.54*		
iv. ZRT ~ Block + Age + (1|Subset)		metric	linear	logarithmic
v. ZRT ~ Block + Age + Block:Age+ (1|Subset)	v. vs vi.	RMSE	**0.955**	0.957
vi. ZRT ~ Block + log(Age) + (1|Subset)		R^2 (marginal)	0.131	**0.135**
		R^2 (conditional)	0.168	**0.186**

Table S24. Full model output for Won and ZRT response variables in the extended version of the Stroop task. In bold the selected models and the best metric in each case.

ZWon

Models		Comparison			
		i. vs ii.	***LR: 18.83, p-value: 1.43 e-5***		
i.	*ZWon ~ 1 + (1∣Subset)*	i. vs iii.	***LR: 51.07, p-value: 8.90 e-13***		
ii.	*ZWon ~ Age + (1∣Subset)*				
iii.	*ZWon ~ Block + (1∣Subset)*	iii. vs iv.	***LR: 19.11 , p-value: 1.233 e-5***		
iv.	*ZWon ~ Block + Age + (1∣Subset)*	iv. vs v.	*LR: 3.32 , p-value: 0.068*		
v.	*ZWon ~ Block + Age + Block:Age + (1∣Subset)*		metric	linear	logarithmic
vi.	***ZWon ~ Block + log(Age) + (1∣Subset)***	iv. vs vi.	RMSE	0.844	**0.844**
			R^2 (marginal)	**0.303**	0.303
			R^2 (conditional)	0.319	**0.324**

ZRT

Models		Comparison			
		i. vs ii.	***LR: 8.33, p-value: 3.88 e-3***		
i.	*ZRT ~ 1 + (1∣Subset)*	i. vs iii.	***LR: 6.08, p-value: 1.36 e-2***		
ii.	*ZRT ~ Age + (1∣Subset)*	i. vs iv.	***LR: 5.89, p-value: 1.51 e-2***		
iii.	*ZRT ~ Block + (1∣Subset)*		metric	linear	logarithmic
iv.	***ZRT ~ Block + Age + (1∣Subset)***		RMSE	**0.936**	0.937
v.	*ZRT ~ Block + log(Age) + (1∣Subset)*	iv. vs v.	R^2 (marginal)	**0.161**	0.156
			R^2 (conditional)	0.200	**0.233**

3.3.3. *Composite cognitive performance score*

Table S25. Parameters for the linear mixed model with the composite cognitive performance score as the outcome and Age (cont) as predictors.

Cognitive performance composite score – school age (discrete)

Predictors	Coefficient	Std. Error	z	P>∣z∣	[0.025	0.975]
(Intercept)	-1.968	0.168	-11.692	**<0.0001**	-2.298	-1.638
School age	0.364	0.031	11.690	**<0.0001**	0.303	0.425
Group Var	0.027	0.015				
Observations	1148					
Groups	26					
RMSE	0.868					
Marginal R^2	0.164					
Conditional R^2	0.193					

brain sciences

Table S26. Parameters for the linear mixed model with composite cognitive performance score as the outcome and Age (continuous) as predictors.

Cognitive performance composite score – age (continuous)						
Predictors	Coefficient	Std. Error	z	P>|z|	[0.025	0.975]
(Intercept)	-1.975	0.162	-12.198	**<0.0001**	-2.293	-1.658
Age (continuous)	0.334	0.027	12.449	**<0.0001**	0.281	0.386
Group Var	0.022	0.014				
Observations	1085					
Groups	26					
RMSE	0.854					
Marginal R^2	0.194					
Conditional R^2	0.218					

References

1. Rueda MR, Fan J, McCandliss BD, Halparin JD, Gruber DB, Lercari LP, Posner MI. Development of attentional networks in childhood. Neuropsychologia 2004; 42:1029–1040.
2. Davidson MC, Amso D, Anderson LC, Diamond A. Development of cognitive control and executive functions from 4 to 13 years: Evidence from manipulations of memory, inhibition, and task switching. Neuropsychologia 2006; 44:2037–2078.
